# speaq 2.0: A complete workflow for high-throughput 1D NMR spectra processing and quantification

**DOI:** 10.1371/journal.pcbi.1006018

**Published:** 2018-03-01

**Authors:** Charlie Beirnaert, Pieter Meysman, Trung Nghia Vu, Nina Hermans, Sandra Apers, Luc Pieters, Adrian Covaci, Kris Laukens

**Affiliations:** 1 Adrem Data Lab, Department of Mathematics and Computer Science, University of Antwerp, Antwerp, Belgium; 2 Biomedical Informatics Network Antwerp (biomina), University of Antwerp, Antwerp, Belgium; 3 Department of Medical Epidemiology and Biostatistics, Karolinska Institutet, Stockholm, Sweden; 4 Natural Products & Food Research and Analysis (NatuRA), Department of Pharmaceutical Sciences, University of Antwerp, Wilrijk, Belgium; 5 Toxicological Center, Department of Pharmaceutical Sciences, University of Antwerp, Wilrijk, Belgium; Hebrew University of Jerusalem, ISRAEL

## Abstract

Nuclear Magnetic Resonance (NMR) spectroscopy is, together with liquid chromatography-mass spectrometry (LC-MS), the most established platform to perform metabolomics. In contrast to LC-MS however, NMR data is predominantly being processed with commercial software. Meanwhile its data processing remains tedious and dependent on user interventions. As a follow-up to speaq, a previously released workflow for NMR spectral alignment and quantitation, we present speaq 2.0. This completely revised framework to automatically analyze 1D NMR spectra uses wavelets to efficiently summarize the raw spectra with minimal information loss or user interaction. The tool offers a fast and easy workflow that starts with the common approach of peak-picking, followed by grouping, thus avoiding the binning step. This yields a matrix consisting of features, samples and peak values that can be conveniently processed either by using included multivariate statistical functions or by using many other recently developed methods for NMR data analysis. speaq 2.0 facilitates robust and high-throughput metabolomics based on 1D NMR but is also compatible with other NMR frameworks or complementary LC-MS workflows. The methods are benchmarked using a simulated dataset and two publicly available datasets. speaq 2.0 is distributed through the existing speaq R package to provide a complete solution for NMR data processing. The package and the code for the presented case studies are freely available on CRAN (https://cran.r-project.org/package=speaq) and GitHub (https://github.com/beirnaert/speaq).

This is a *PLOS Computational Biology* Software paper.

## Introduction

1D NMR spectroscopy has been a popular platform since the early days of metabolomics. Although less sensitive than the complimentary and more common LC-MS technology, NMR has its merits. For one, it is an unparalleled technique in determining the structure of unknown metabolic compounds. Furthermore, because it is a non-destructive technique, samples can be reanalyzed later or can be used in a different spectroscopic analysis such as mass spectrometry. Also, an NMR spectroscopy experiment requires little sample preparation compared to LC-MS, thus limited unwanted extra variability is introduced. Finally, the results of an NMR analysis are less dependent on the operator and instrument used. All these factors make 1D NMR spectroscopy a technique with a relatively high reproducibility and rather minimal researcher bias [1]. There are however also drawbacks associated with the technique. First, the aforementioned low sensitivity is an important issue as the dynamic range in real biological samples surpasses the NMR detection range. This is particularly problematic when the goal is to identify an unknown metabolite with a low concentration.

To get the best of both worlds, combining large scale LC-MS analysis with NMR spectroscopy has been presented as an option to yield valuable novel insights in metabolic pathways and biomarkers [2–4]. From a data processing perspective, this combination is not trivial. The data analysis of NMR is not as automated as LC-MS data analysis, which can rely on open-source solutions like XCMS [5]. Most NMR data analyses are still performed with commercial software [6]. While the reproducibility of the NMR experimental techniques is high, the data analysis still requires a substantial degree of user intervention. This results in the possible introduction of bias and lower research reproducibility, meaning that the data analysis can not be easily replicated by others. The absence of standardized and automated NMR metabolomics workflows is the main culprit despite recent progress. See Table 1 for an overview of freely available NMR software. Not all these NMR analysis tools are applicable to all research setups. Some serve only specific purposes like BATMAN [7], for example, which is aimed at obtaining concentration estimates for known metabolites from the raw spectra. However, a lot of untargeted experiments are in search for not only known metabolites, but also unknown ones. These experiments require tools that can process large amounts of data in a scalable way.

**Table 1:** An overview of open source NMR data processing solutions.

Name	Platform	Aim
BATMAN [7]	R	Estimating relative metabolite concentrations from a list of target metabolites and quantifying individual metabolites by modelling the resonances in the NMR spectra. As it requires a target list of metabolites it is less suited for fully untargeted approaches.
Bayesil [8]	web	Automatic quantification and identification of metabolites from a reference list. This method starts from the raw NMR free-induction-decay signal and thus does not require conversion to the NMR spectra, however only certain formats are supported and the data have to be collected in a specific way.
ChemoSpec [9]	R	A general package for processing of spectroscopic signal (NMR, infrared or Raman) aimed at metabolomics. Uses the binning approach for spectra summarisation and small shift correction. Also incorporates the CluPA algorithm for raw spectral alignment from speaq (v1.0—1.2.3)
MetaboAnalyst [10–12]	web	Arguably one of the most widely used platforms for metabolomics analysis. It has specific modules for biomarker analysis, pathway analysis, statistics, etc. For NMR data however it only accepts binned data or peak data which has been grouped over the samples (so effectively a matrix with samples and features). Therefore our method can be used to provide a starting point for an analysis with MetaboAnalyst.
muma-R [13]	R	A package for data (pre-)processing and statistics aimed at MS and NMR metabolomics data. As it requires binned NMR data or NMR peak data it can also be used in combination with speaq 2.0 to provide a high quality peak list as a starting point.
MVAPACK [14]	GNU Octave	A toolbox with a large number of capabilities for reproducible NMR data analysis starting from the raw NMR Free-induction-decay signal. Also relies on the binning method for spectra summarization.
speaq (v1.0—1.2.3) [15]	R	The CluPA algorithm for raw spectral alignment was the main object of speaq v1.0—1.2.3. It can also be used to quantify spectral regions that differ between case and control classes.
specmine [16]	R	A package for metabolomics data analysis. The available methods are a collection from other well known metabolomics packages and some general methods from multivariate statistics and machine learning.

A typical workflow for NMR spectral analysis consists of several pre-processing steps, such as baseline correction, raw spectral alignment, spectra summarization and grouping. This is then followed by statistical analysis. The spectra summarization step and the alignment/grouping step are the most time consuming. Spectra summarization is the transformation of all the experimental measurement points into a small number of features, which are more suited for automated analysis. Multiple spectra summarization techniques exist, each with their own advantages and drawbacks [17]. The specific method that is chosen can result in user-introduced bias and low reproducibility. This is the case for the most commonly used summarization approach: the so-called binning or bucketing method [18]. This method was introduced to compensate for small spectral shifts between samples. It allows to vastly reduce the number of measurements points to a limited number of variables (the bins) in one single, automated step [19]. There are however major drawbacks to this method that have a profound influence on the results [20]. In particular, it is not straightforward to define the boundaries of the bins in crowded spectra. Automating this process may lead to splitting up small but relevant peaks. Manually checking the bins on the other hand is extremely tedious and tweaking the boundaries can forfeit any attempt for reproducibility. Several methods have been proposed to tackle the bin boundary issue [21–23], but this is not the only concern. Loss of information is intrinsically linked to the binning approach as entire bins are simply summed together.

At the end of an analysis based on the binning approach, when several bins are found to be interesting, it is still necessary to go back to the raw spectra to manually check the intervals. This is necessary to find the ppm values of the actual peaks of interest that can then be used to query a database, like HMDB [24]. This introduces yet another point where user intervention is required, which slows down the whole process and hampers the use of an automated workflow.

In this paper, we present the speaq 2.0 method. The underlying core paradigm is to efficiently summarize spectra with little user interaction, high speed and most importantly little loss of information whilst greatly reducing the dimensions of the data. The overall aim however, is not to construct yet another all-encompassing package for NMR analysis, but more importantly, to construct a method that can complement established tools for NMR data analysis like MetaboAnalyst [12], by improving performance, analysis quality and reproducibility. This is achieved by improving the quality of the peak lists which are the starting points for MetaboAnalyst [12] or muma-R [13]. By automating the important peak picking step in the NMR analysis workflow, less researcher bias is introduced hereby greatly improving reproducibility. The automation potential of the package makes it suitable for the fast analysis of NMR spectra in a way that is very comparable to how LC-MS spectra are analyzed. In the future, this method could be effective for high-throughput analyses in which LC-MS and NMR data are combined to achieve better results. Nonetheless, a complete standalone analysis pipeline is presented with the focus on user-friendliness. This is to allow also non-expert users to be able to work with open-source tools instead of the black-box proprietary software.

The basic proposition of speaq 2.0 is to use wavelets to summarize the peaks within the spectra. By working with the peak data instead of the raw spectra or binned spectra a great reduction in data size can be achieved without a large loss of information. The Mexican hat wavelet is used to mathematically represent the peaks with only a few values instead of the tens or hundreds of raw data points describing the peak in the original spectrum. Besides the data reduction, the additional advantage of using wavelets is that the need for baseline correction and smoothing is eliminated with no loss of sensitivity or increase in false positives [25, 26].

## Design and implementation

### Workflow

**Fig 1:**
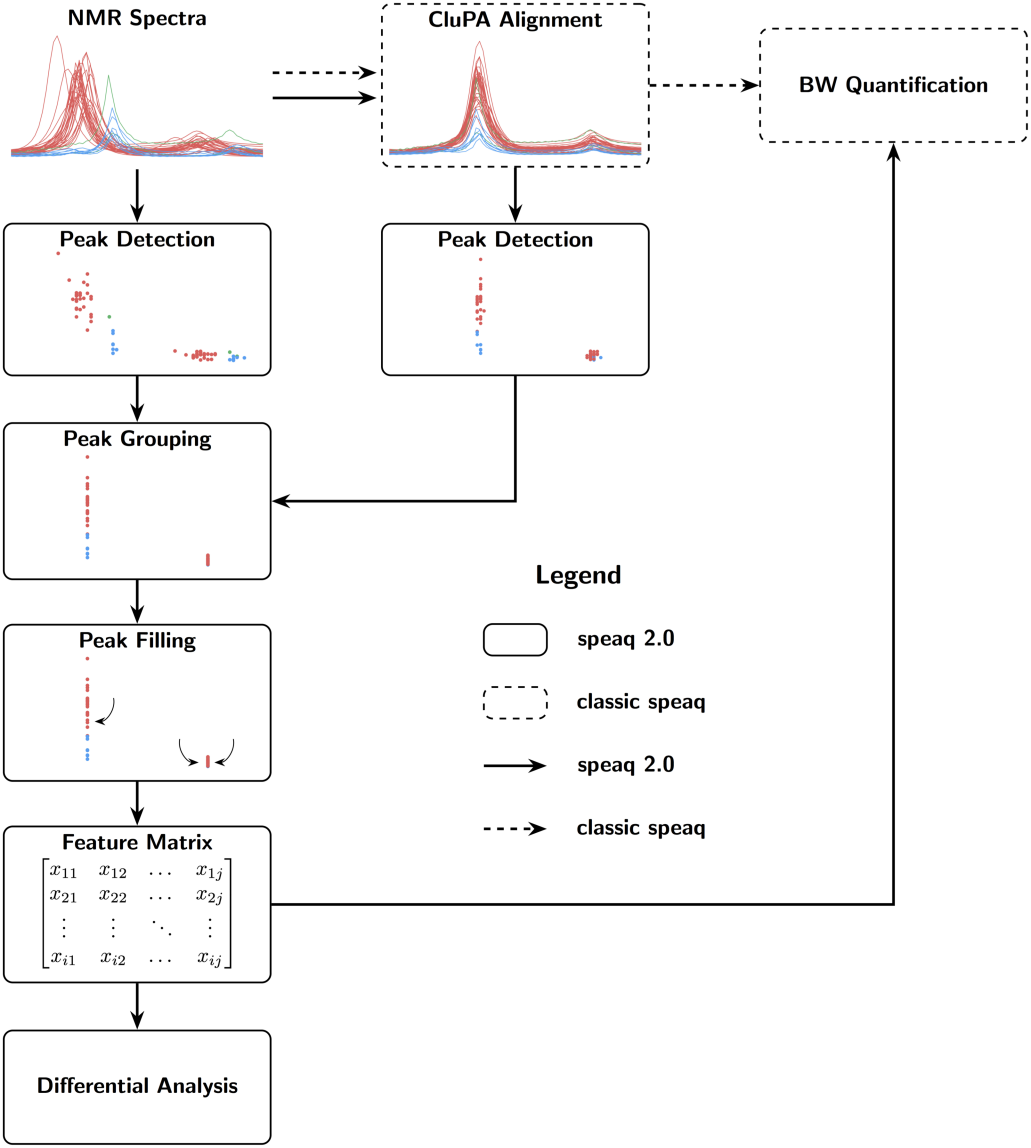
Possible workflows of speaq 2.0. The newly presented methods are standalone (full black arrows) or can be used together with the CluPA alignment algorithm and BW quantification method that were made available in the first speaq implementation (v 1.0—1.2.3) [15] (dashed arrows). It is still possible to perform an analysis based on raw spectra alone, as per the classic speaq (v 1.0—1.2.3) analysis. With the new methods, raw data is converted to peaks, and every peak is summarized with ppm location and width, intensity and SNR. These peaks are subsequently grouped and optionally peak filled (missed peaks in samples are specifically searched for). The resulting data is converted to a feature matrix that contains intensities for each peak and sample combination. This matrix can then be used in statistical analysis with built-in or external methods.

The NMR data analysis workflow of speaq 2.0 is depicted in Fig 1. Spectra serve as input, then peak picking with wavelets is applied to transform the spectra to peak data. These peaks are then grouped into features with the grouping algorithm and peak filling is applied to fix missing values. The resulting matrix of features and samples is then used in statistical analysis. This approach is intrinsically different from the one available in the original speaq (v1.0—1.2.3), which was centered around the concept of aligning spectra with the CluPA algorithm. The BW-quantitation function of speaq v1.0—1.2.3 allows users to find regions that are different between two groups, such as case and control. However, speaq v1.0—1.2.3 does not support the case of multiple groups or ratio variables. Also, it requires a binning step to obtain a feature matrix to be used in pattern mining or statistical approaches such as PCA. The new speaq 2.0 methods allow multiple groups and circumvent the need for binning. Overall, speaq 2.0 offers a novel way of processing NMR data, with the option to use the classic spectral alignment techniques as an intermediary step. The entire package is designed to easily and quickly build a reproducible workflow to obtain the peaks that are of interest to the experiment. Although the approach is very different to the one available in speaq v1.0—1.2.3, the choice was made to integrate the functionality of both speaq v1.0—1.2.3 and speaq 2.0 in one single package. The main benefit is that the methods are fully compatible and it allows existing speaq users to easily extend their workflows as needed. The individual steps of the speaq 2.0 approach are described in more detail in the following section.

### Pre-processing steps

The input to the workflow consists of spectra in the intensity (y-axis) vs ppm (x-axis) format. This means that the free induction decay (FID) signal coming from the NMR spectrometer has to be converted to spectra by using the Fourier transform. In addition, before peak picking, the spectra can be aligned with the included CluPA algorithm [15] (the core of speaq v1.0—1.2.3). Note that it is also possible to analyse spectra that have already been aligned with other methods like icoshift [27]. However, depending on the algorithm used, aligning raw spectra can result in the distortion of small peaks [28].

#### Peak detection: From spectra to features via wavelets

The Mexican hat wavelet is used to perform the peak detection. It is a suitable wavelet because it resembles a peak by being symmetrical and containing only 1 positive maximum [25]. This peak detection method was inspired by the CluPA alignment algorithm [15] where wavelets are used to find landmark peaks to aid in the alignment. The interaction with the wavelets relies on the MassSpecWavelet R package which performs the actual peak detection as per the method outlined by Du et al [25]. A spectral segment (an intensity vector) containing a peak is converted to wavelet space by changing the scale and position of the mother wavelet and obtaining the wavelet coefficient for each combination of scale and position. The wavelet coefficient can be seen as a metric for how well the wavelet matches the peak. The entire intensity vector is converted into a wavelet coefficient matrix. This wavelet coefficient matrix is where the actual peak detection takes place. A ridge (line of high values) will appear in the matrix where the position of the wavelet matches the position of a peak in the spectrum. The height of the ridge is not constant as it varies with the scale of the wavelet. At the point where the scale best matches the width of the peak in the spectrum the ridge will feature a local maximum. The problem of peak finding is thus shifted to finding ridges, and finding the maximum of each ridge. See Du et al. for more details [25]. The result is a peak detection that is both sensitive to low and high intensity peaks and insensitive to background noise (as noise will not produce a noticeable ridge).

Although the default parameters of the peak picking approach work for most NMR experiments, different parameters for the peak picking can be set according to user preferences. For example, the baseline intensity threshold (to focus on higher peaks only, default is 1000), the signal-to-noise ratio threshold and the scales to be used for the wavelets. Note that the default parameters are set up for untransformed spectra, when spectra are scaled to max intensity 100 or 1 different settings may be more appropriate. All data sets were analysed with the default speaq 2.0 values.

After the peak detection, the spectra (intensity vs ppm data) are converted to a dataset with peakIndex and peakValue values. Note that this peakValue vs peakIndex dataset has a substantially lower dimension than the original data. The peakIndex is directly linked to the ppm value. The peakValue is related to the wavelet coefficient that describes the peak. This wavelet coefficient is an approximation for the area under the peak curve and this used throughout the analysis. Since peak height is of interest for some NMR data analysis pipelines, the option to work with peak heights has been made available in the peak picking function.

#### Peak grouping

**Fig 2:**
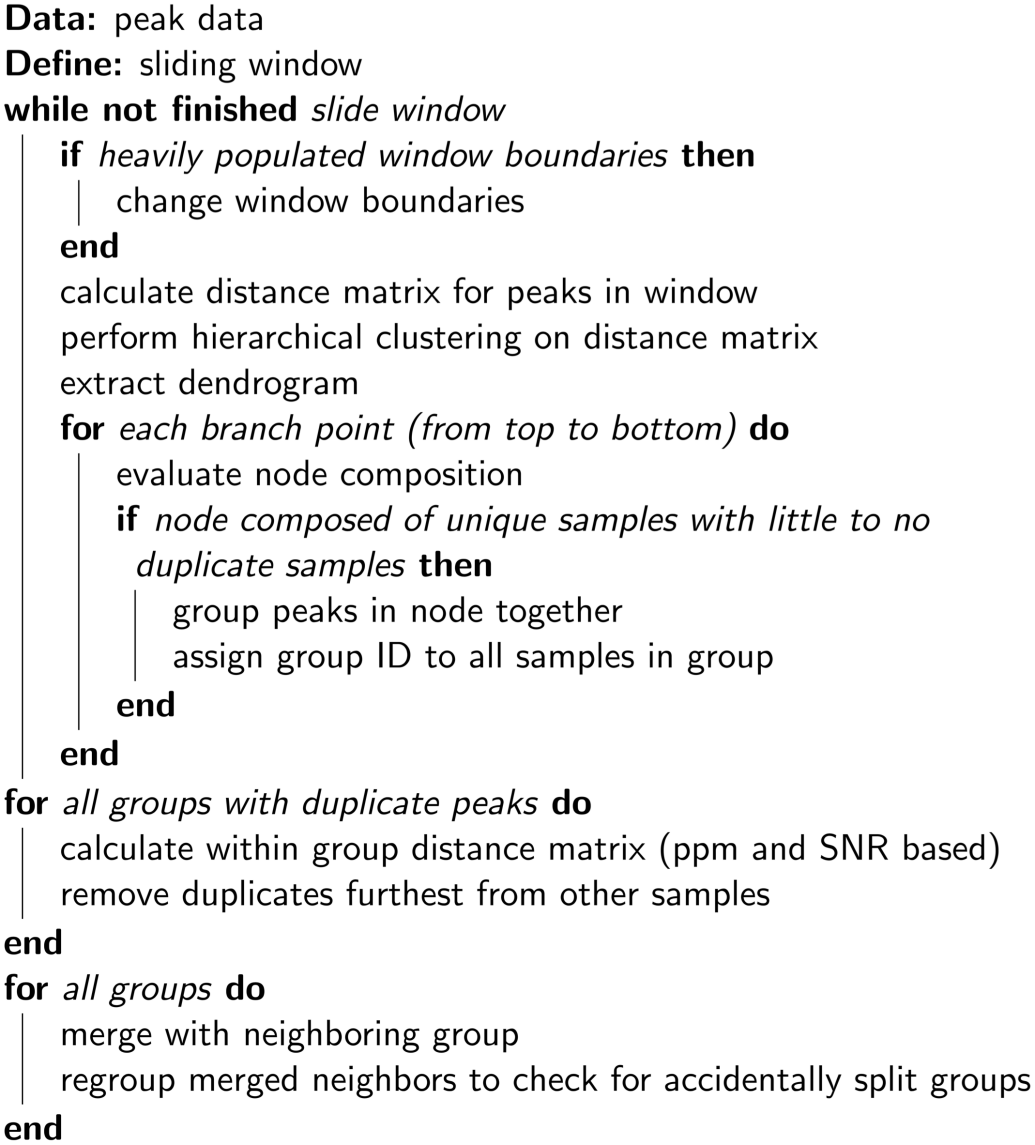
Grouping algorithm pseudocode.

The peaks resulting from the wavelet peak detection are not perfectly aligned since no two peaks are exactly the same and different spectra can be shifted relative to each other. These shifts can be caused by differences in sample environment (pH, solvent, etc.) or differences in experimental conditions (temperature, magnetic field homogeneity). However, the aim is to go towards a feature dataset whereby a feature is defined as a group of peaks with at most one peak per sample belonging to that feature. This means the peaks have to be grouped with a single index value describing the group center. To group the NMR peaks we can make optimal use of the results of the wavelet based peak detection step. Not only ppm values but also signal-to-noise ratio and sample values can provide information to aid in the grouping. The hierarchical clustering based algorithm developed for grouping does not require a reference sample as it divides the samples in groups based on the Gower coefficient [29]. The merit of the Gower distance is that variables of different units (here ppm and intensity) can be safely used together. It is calculated by normalizing each variable to a value between 0 and 1. The distance between two data points is then the sum of the distances for each variable. As such the Gower distance can be seen as a Manhatten distance on normalized data. The grouping algorithm’s pseudocode is displayed in Fig 2. A more detailed description can be found in S1 Appendix.

Note that this method is designed to process data that is sufficiently well aligned. If this is not the case the method will most likely underperform because of the larger overlap between peaks. Nonetheless the method even works on data with non-trivial shifts between samples as is the case in the wine benchmark dataset [27]. Extremely shifted spectra can be aligned with existing methods such as CluPA [15], prior to peak detection.

#### Peak filling

The purpose of peak filling is to detect peaks that may have been missed in the first round of peak detection. To illustrate this problem, we can think of a scenario in which, for example, the user sets a high intensity threshold for peak detection. The features matrix will then be composed of features that correspond to locations with high peaks. If certain samples have low peaks in this region the peak filling step can be used to find these peaks, because peak filling works without an intensity threshold. This reduces the amount of missing data in the feature matrix. In an other scenario, peaks can be deliberately deleted if the grouping algorithm detects two peaks from the same sample in the same group. If this peak actually belonged to a different group it can then be recovered with the peak filling step. For each feature, the peak filling algorithm will specifically search the raw data for peaks of missing samples. A small section of the missing sample spectra is used to perform the peak detection. This small section is of length 512 measurement points (small) or 1024 (large), as this greatly speeds up the computation of the Fourier Transform used by the MassSpecWavelet package [25]. A more refined wavelet search is performed in this region starting from the average group values for peak location and width. If a peak is found it has to be within a set distance from the group. The default is 10 measurement points, which is approximately between 0.001 ppm and 0.01 ppm, depending on the NMR instrument settings. This distance is small as otherwise distant peaks could be assigned to the group. If no peak is found then it is still regarded as a missing value and can be imputed later. The end result is more peaks, which in turn results in a more robust statistical analysis afterwards as less missing values have to be imputed.

### Statistical analysis

Following peak filling, the data can now be represented in the form of a matrix with samples for rows, features (peak groups) for columns and peak values in each matrix cell. Each of these peak values corresponds to the size of the original peaks as quantified by the wavelets. A huge number of techniques for univariate and multivariate statistics (e.g. PCA, PLS-DA) and machine learning (e.g. SVM, random forest) can be applied to this data matrix. Most of these methods are made available through different R packages which can be found on CRAN, Bioconductor [30] or Github. The output provided by speaq is compatible with the majority of these methods, as most of these allow to submit a data matrix and a response vector (class labels) as input. A selection of methods useful for statistical analysis have been directly integrated into the speaq 2.0 framework: a tool to perform scalings, transformations and imputations and a differential analysis method.

#### Scaling and imputation

Before statistical analysis methods like PCA can be used, the missing values in the data have to be imputed. This step is often done in tandem with the desired scaling method since otherwise data can artificially be created. For example, imputing zeros followed by z-scaling is not the same as z-scaling followed by imputing zeros. The latter actually corresponds with imputing mean values. For all benchmark datasets zeros (the default) are used for imputing missing peak values in the data matrices as this indicates a non present peak. Although other methods are available, for example kNN imputation [31] and random forest based imputation [32].

After imputation, the optional normalization and scaling steps are executed. Several methods commonly used in metabolomics are available such as Probability Quotient Normalization (PQN) and Pareto scaling [33]. Pareto scaling is most suited for metabolomics since it reduces the effects large signals while keeping the data structure roughly intact. It is governed by the formula in Eq (1) with **y**_*j*_ the *j*^*th*^ feature vector containing the peak values *y*_*i*,*j*_ of all samples 1 … *i* … *N* and *σ*_*j*_ the standard deviation of **y**_*j*_.

$$\begin{array}{c} {\tilde{y}}_{j}=\frac{y_{j}}{\sqrt{\sigma_{j}}} \end{array}$$ (1)

#### Differential analysis

A differential analysis method based on linear models is available in speaq 2.0. This function provides a way of identifying significant features with (adjusted) p-values. Specifically, for each feature 1, …, *j*, …, *K* consisting of peak values *y*_*i*,*j*_ of samples 1 … *i* … *N* a linear model of the form

$$\begin{array}{c} y_{j}=x\;\beta_{j}+\varepsilon \end{array}$$ (2)

is constructed with **x** the response vector (N elements, for example class membership, a bioassay result, etc.), **y**_*j*_ the *j*^*th*^ feature vector and ***ε*** the vector of errors *ε*_*i*_. Now for each *β*_*j*_ we can test whether there is a significant relationship between feature **y**_*j*_ and **x** by testing the hypothesis that *β*_*j*_ = 0 (two-tailed t-test). The *K* p-values can be used to find peaks significantly associated with the response vector. Several multiple testing corrections are available within the speaq 2.0 framework. While the default is Benjamini-Hochberg, for the purposes of this study, the stringent Bonferroni correction was applied to all reported p-values. Note that in the case of only two classes, this method is equivalent to the t-test.

### Metabolite identification

After statistical analysis the relevant peaks can be matched with the molecules responsible for these peaks. Several databases with NMR metabolomics data are available [17]. One of the more user friendly ones is the Human Metabolome Database (HMDB) [24], as it allows to search for compounds by providing the ppm values of the peaks of interest. To obtain the metabolites for the onion intake in mice data the latest version of HMDB (3.6 [34]) was used. It is however not optimal to submit all significant peaks in a single query to this database. The reason for this is that HMDB works by matching the queried peaks to the database and then sorting the matched molecules according to their Jaccard index. For two sets the Jaccard index is the number of common elements (the intersection), divided by all the elements (the union), or in this specific case the number of matched peaks divided by all peaks in the query. Thus, when submitting all peaks at once we risk not finding the correct metabolite as adding additional peaks from molecule B when trying to identify molecule A will dilute the results. To reduce this effect a correlation analysis can be performed to indicate which peaks belong together. The underlying assumption is that NMR spectra peaks originating from the same molecule exhibit similar behavior over all samples. Therefore the peaks that correlate strongly with each other are most likely to come from the same metabolite. The speaq 2.0 output format is compatible with the R functionality for correlation analysis. The correlation matrix is visualized with the corrplot R package [35] which clusters the peaks according to their correlation. The number of clusters are chosen by the user between one and the total number of peaks. The correlation within each cluster is affected by the chosen number of clusters. The user is responsible for choosing this number of clusters and evaluating the corresponding performance. After the correlation analysis step, the ppm values of peaks in a correlated cluster can be submitted directly to HMDB via a built in speaq 2.0 function (HMDBsearchR, note this will open a webpage). This produces a list of metabolites ordered by Jaccard index. It is up to the user to determine which Jaccard index is to be considered high enough.

### Benchmark data

To validate the presented approach three datasets are analyzed: one simulated dataset for which the ground truth is known and two publicly available datasets which have been analyzed in published studies.

1. The wine dataset by Larsen et al. [36] consists of 1H NMR spectroscopy data of 40 table wines (red, white & rosé). The focus of Larsen et al. was not to investigate differences between wines of different colour and origin, but merely to evaluate how pre-processing methods like alignment and interval selection can aid in chemometrics and quantitative NMR analysis [36]. Wine is a good example for evaluating alignment algorithms because of the often substantial differences in pH, which can cause large shifts in the NMR spectra. Because of this property, the wine dataset has been used to evaluate the performance of several alignment algorithms, like COW [36], icoshift [27] and CluPA [15].
2. The simulated dataset is constructed by combining the 1H NMR spectra of two metabolites, namely 3-Hydroxyphenylacetic acid (HMDB0000440) and 3,4-Dihydroxybenzeneacetic acid (HMDB0001336). The NMR spectra of both metabolites can be downloaded from the Human Metabolome Database [34]. The dataset consists of 20 simulated spectra that are combined in such a way as to include variation that is comparable to the most common between-sample variation found in NMR spectra. Most notably, there is variation in peak height, peak location, and peak composition. The variation in peak composition is caused by both metabolites having peaks at almost identical locations. This results in two sources of variation in peak location namely, the variation introduced by the random shift left or right and by the mixing factor that describes the weight of each metabolite in each spectrum. See S2 Appendix for more details about how this dataset was generated.
3. The onion intake in mice dataset originates from a nutri-metabolomics study by Winning et al. [37]. The objective of the study was to search for onion intake biomarkers. The underlying idea was that if their workflow can identify biomarkers for onion intake, it could also be used to locate biomarkers in other studies. 32 rats were divided into 4 categories each receiving a specific onion diet: control (0% onion), 3% onion residue, 7% onion extract and 10% onion by-product. Urine samples were collected during 24 hours and analyzed with proton NMR spectroscopy to characterize the metabolome of the different onion fed mice. More details can be found in [37].

Both the wine and onion datasets were made available by the University of Copenhagen at http://www.models.life.ku.dk/.

## Results

### Wine data

The first public validation dataset concerns the NMR spectra of table wines. This dataset has been often used to evaluate algorithms to align raw spectra. The new speaq 2.0 workflow which transforms spectra to peaks, which are then grouped together, can also be used to process this dataset.

#### The peak based approach for data reduction

By using the speaq 2.0 peak picking method, followed by grouping and peak filling, the size of the data is greatly reduced. This is done in multiple steps. First, peak detection is applied to the raw spectra to convert the large raw measurement data matrix of 40 samples by 8712 measurements to a smaller matrix of 6768 peaks by 6 columns consisting of values describing the peaks. The data reduction after this step does not seem overwhelmingly large. However, it is important to realize that this is only a reduction in redundant information which is accompanied with little loss of information thanks to the wavelets. Furthermore, most of the correlation between consecutive measurement points in the spectral data is removed. Next, the peaks are grouped, resulting in the same dataset as the peak data, but now each peak has been assigned to a group. Such a group consists of a collection of peaks with at most one peak per sample. This grouped peak data can now be represented as a matrix, with groups as columns, samples as rows, and peak intensities as the matrix elements. The true data reduction becomes apparent now as there are only 207 peak groups, which correspond to the features used in further analysis. The original matrix of 40 by 8712 is thus converted to a matrix of 40 by 207.

#### From feature matrix to locating differences between spectra

**Fig 3:**
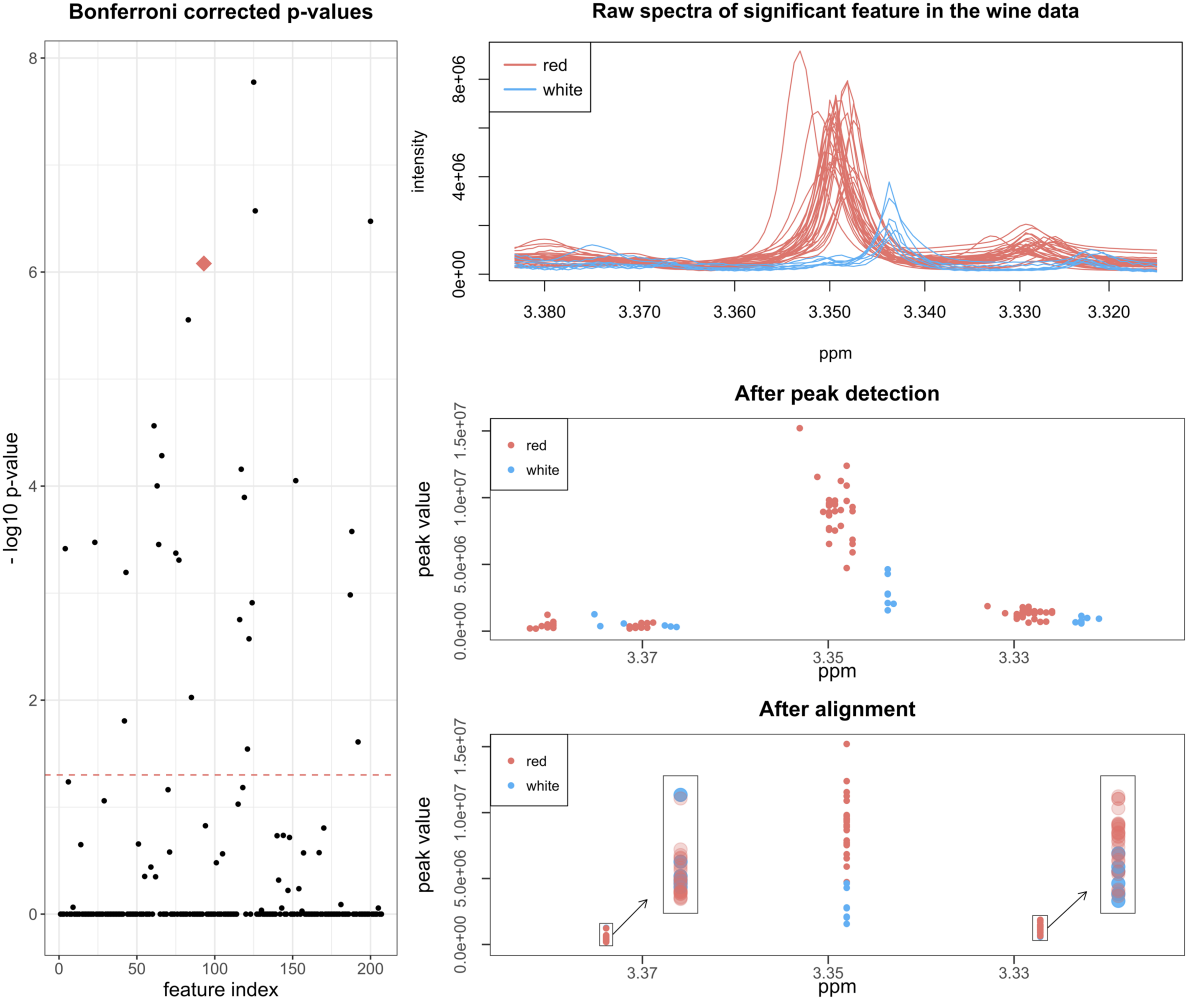
Visualization of Bonferroni corrected p-values. Numerous features have a corrected p-value below the significance threshold of 0.05 indicating that there is a significant difference between red and white wine. An example of a significant feature (indicated with the red diamond) is represented on the right with its raw spectra (top), the data after peak detection (middle) and the data after grouping (bottom).

We can locate those features that are associated with wine type. Before any analysis the data matrix is Pareto scaled and centered. The first step in a multivariate analysis is often principal component analysis (PCA). The results show that there is a clear difference between on one side red and on the other white and rosé wines (S1 Fig). However, a differential analysis method incorporated into speaq 2.0 can be sued to investigate the specific features that are different between the red and white wine classes. The results of the differential analysis is a series of p-values, one for every feature, which indicate how useful each feature is in building a linear model that can discriminate between the two wine classes. The p-values are displayed in Fig 3 along with the raw spectra and grouped peak data for one of 33 significant features. When looking at the spectra that correspond to these features, the difference between red and white wines is obvious. However, manually searching the original spectra for these difference regions would be extremely tedious and time consuming. With speaq 2.0, this entire process takes about 3 minutes with 1 CPU and a mere 50 seconds with 4 CPUs (2.5 GHz machine).

#### Comparing peak grouping to raw spectral alignment

**Fig 4:**
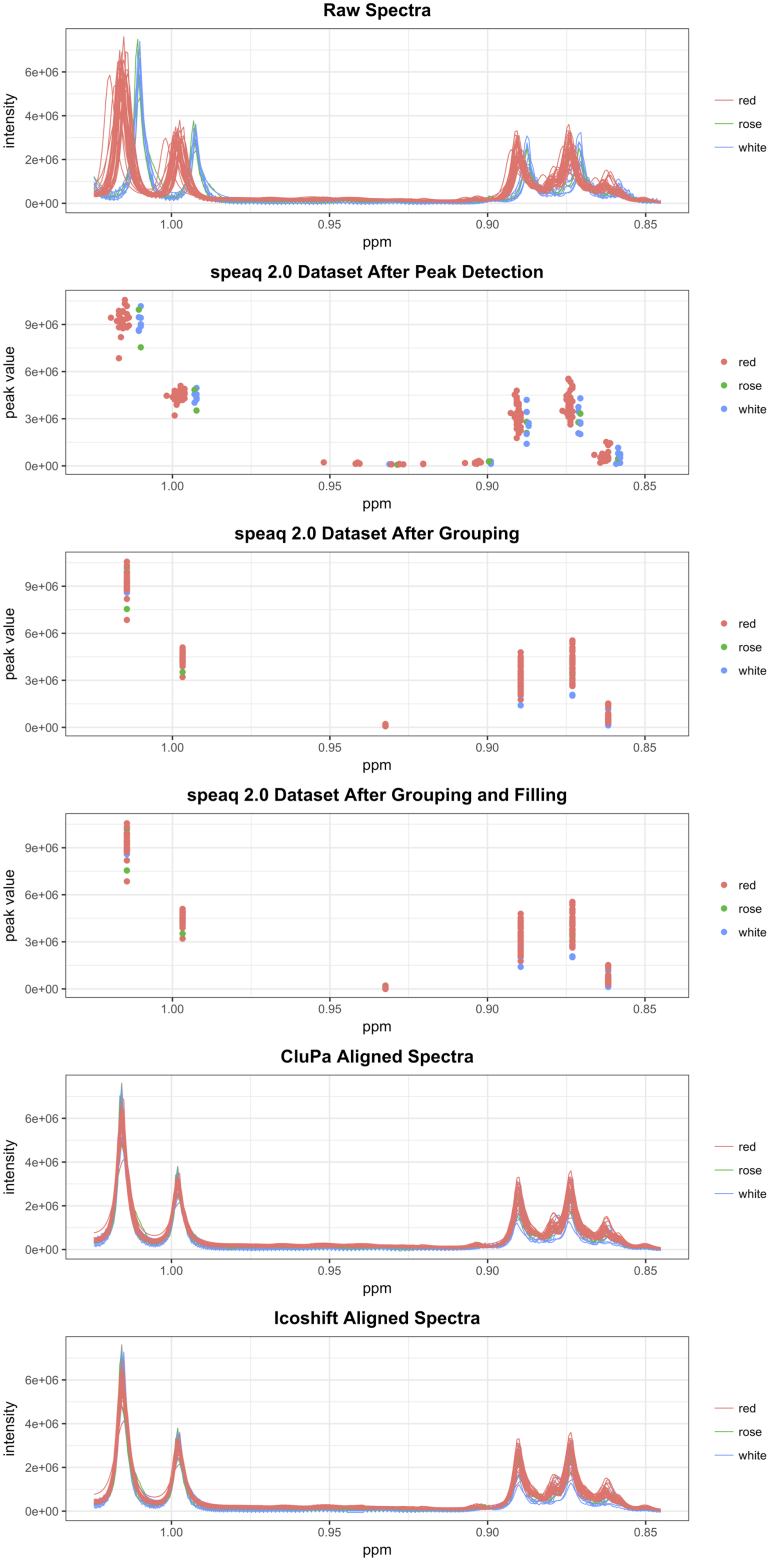
Raw spectral alignment methods and peak based grouping methods perform equally. When the peak shifts between samples (caused by pH differences etc.) are less than the distance between adjacent peaks, all methods perform as expected. The raw spectra based methods (CluPA from the speaq v1.0—1.2.3 and icoshift) mitigate the differences in peak shifts and the peak based method groups the peaks accordingly.

The new speaq 2.0 approach differs from spectral alignment algorithms, such as CluPA and icoshift [15, 27], but the final results should correspond to each other: grouped peaks should correspond to aligned peaks in the spectrum. By comparing the results from each, we can study the cases where the peak based method performs better, equal or worse compared to the raw spectra based methods. The performance of both types of spectra processing methods (peaks vs raw spectra) is dependent on the content and specifics of the spectra. Most notably the number of peaks and the shifts between sample spectra (caused by pH differences etc.) largely dictate how well these methods will perform. Generally, if the peak shifts between samples are less than the distance between consecutive peak groups, all methods perform as expected. An example of this can be found in Fig 4. Beyond these ideal cases, several alignment or grouping mistakes can occur. The following illustrates a small portion of issues that can arise when processing 1D NMR spectra.

1. Many peaks are in a small region causing overlap and there is no clear indication as to which peaks correspond to each other. A clear example of this situation is depicted in S2 Fig. The speaq 2.0 grouping method based on peaks performs similarly to the other methods and in some cases even provides superior grouping solutions (although there is no real way to say which of the smaller peaks belong together). The reason is that it sees all peaks and tries to group them locally. This is in contrast to the CluPA algorithm from the original speaq which only regards the landmark peaks and aligns the highest ones in this crowded region, but the spectra are clearly overshifted. The icoshift algorithm provides a better solution than CluPA in this case but the results remain suboptimal.
2. A single sample shows unique behavior compared to all other samples. An example of such a situation is depicted in S3 Fig. In this case no method performs as it should and every method introduces errors or artifacts. It is however important to note that such unique cases will usually not show up in the final statistical analysis since these analyses often focus on general group differences and are robust against outlier samples.
3. The shift between samples is larger than the difference between two adjacent peaks in a non crowded peak region. An example is shown in S4 Fig. Both the raw spectra approaches (CluPA and icoshift) align the spectra as expected. The speaq based approach initially groups peaks wrongly. However, this wrong alignment can be detected by using a built in function of the speaq package which calculates the silhouette values for each group (see S3 Appendix for definition and implementation). Groups that are flagged as having bad silhouette values are regrouped. After this step the results correspond to those of the correctly aligned spectra.

### Simulated dataset

**Fig 5:**
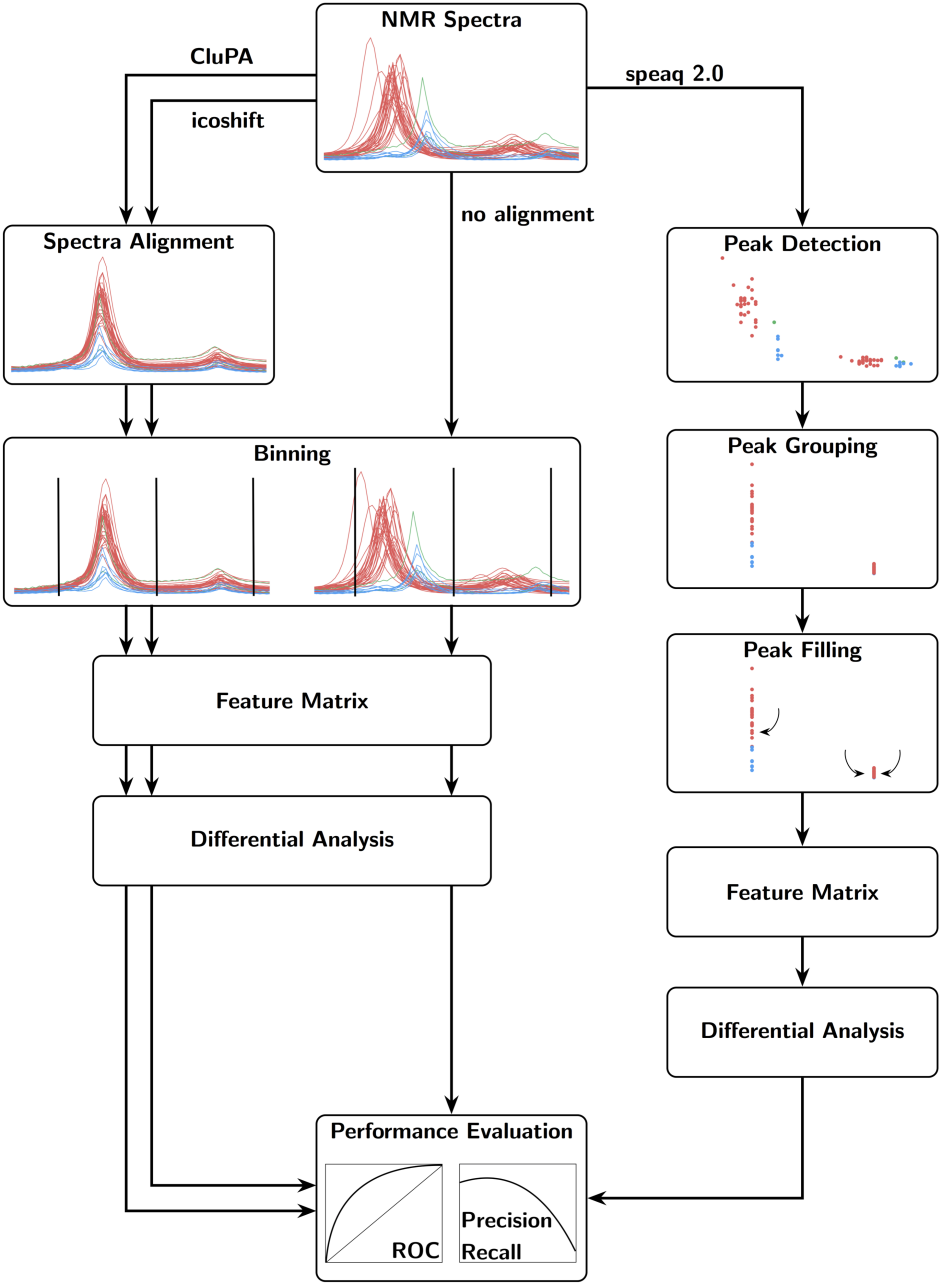
Performance comparison workflow. The default way of processing 1D NMR spectra is illustrated on the left. The case vs. control spectra are aligned and are then binned to produce features which can be used in statistical analysis. Note that the spectral alignment step can be skipped as the binning approach can correct for small shifts. This default processing approach is compared to our method shown on the right. The aim of both methods is to point the user to the peaks/intervals that discriminate between the two groups.

**Fig 6:**
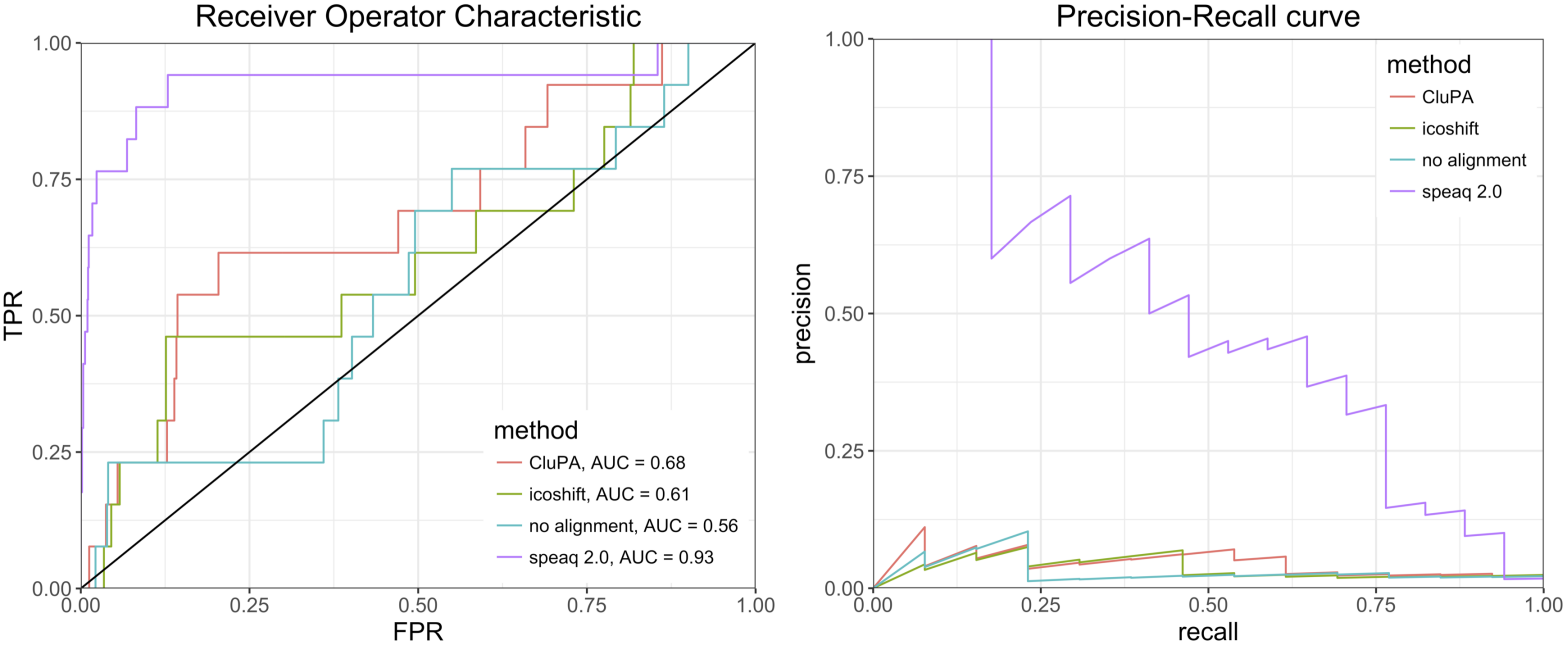
Performance comparison with ROC and P-R curves on a simulated dataset. Binning raw unaligned spectra results in the worst performance. The two alignment tools (CluPA and icoshift) show an increase in performance compared to no alignment but are still hampered by the binning step. The new speaq 2.0 workflow has the highest performance on the ROC and P-R curve.

To actually quantify the performance of the new speaq 2.0 approach, we require a dataset for which the ground truth is known. For this purpose a simulated dataset was constructed (see S2 Appendix for details) from the 1H NMR spectra of two different metabolites, with a mixing factor *m*_*i*_ chosen from a bimodal distribution to simulate two different groups. This effectively corresponds to a case vs control study. This simulated dataset is also processed with 2 common NMR spectral alignment methods, namely CluPA [15] and icoshift [27]. It is not straightforward to quantify the performance of speaq vs the alignment algorithms on the same level since the alignment algorithms do not result in a feature matrix. However, we can compare the new speaq 2.0 workflow to the default workflow of alignment followed by binning. The setup is displayed in Fig 5. By binning the aligned spectra we obtain a feature matrix. The spectra are binned by using the functionality in the ChemoSpec R-package [9]. The bin.ratio parameter is set to 200, which equates to binning with a binwidth of 0.018 ppm. The resulting feature matrix is then processed with the differential analysis functionality of speaq to identify the relevant variables that are significantly different between case and control. This results in a p-value for each bin and this output can be compared directly with the output of the peak based approach of speaq via ROC and Precision-Recall curves, see Fig 6. Several observations can be made from these results:

1. The lowest performance is obtained when simply binning the raw shifted spectra. This is to be expected as it is more likely that peaks will be split over multiple bins because of the large shifts.
2. By aligning the spectra before the binning step the performance increases. Although the spectral alignment methods have occasional artifacts, many peaks in the spectra are in fact aligned correctly, thereby aiding in reducing the splitting of peaks over multiple bins. Nonetheless, this approach is still hampered by the binning step.
3. The peak based method of speaq 2.0 performs best on the simulated case vs control dataset. This is predominantly caused by the different way of obtaining features. With the binning approach, features consist not only of the peak signal but also of the adjacent signals, which are often background. This background effectively reduces the difference between two samples, as the background signal is often similar in scale. With the peak picking approach of speaq 2.0, only the peak is used to describe a feature. This results in greater differences between samples and this in turn makes it easier for the statistical approaches to spot the relevant differences.

### Onion intake in mice data

With this validation dataset, we will compare the results of the new speaq 2.0 workflow with those obtained by Winning et al. [37]. This dataset contains onion 4 groups of mice with increasing percentages of onion in their diet (0, 3, 7 and 10%).

#### Towards a small and usable data matrix

The original analysis by Winning et al. [37] used binning to process the spectra. Here we use the new speaq 2.0 workflow to convert the raw NMR spectra (S5 Fig) to peaks (S6 Fig). Next the peaks are grouped, peak filled and converted to a feature matrix. The dimensions of this feature matrix are 31 samples by 677 features. This is a substantial reduction from the original spectra matrix (31 samples by 29001 measurement points). This feature matrix is the input for the following statistical analysis.

#### No group trend is found by PCA

**Fig 7:**
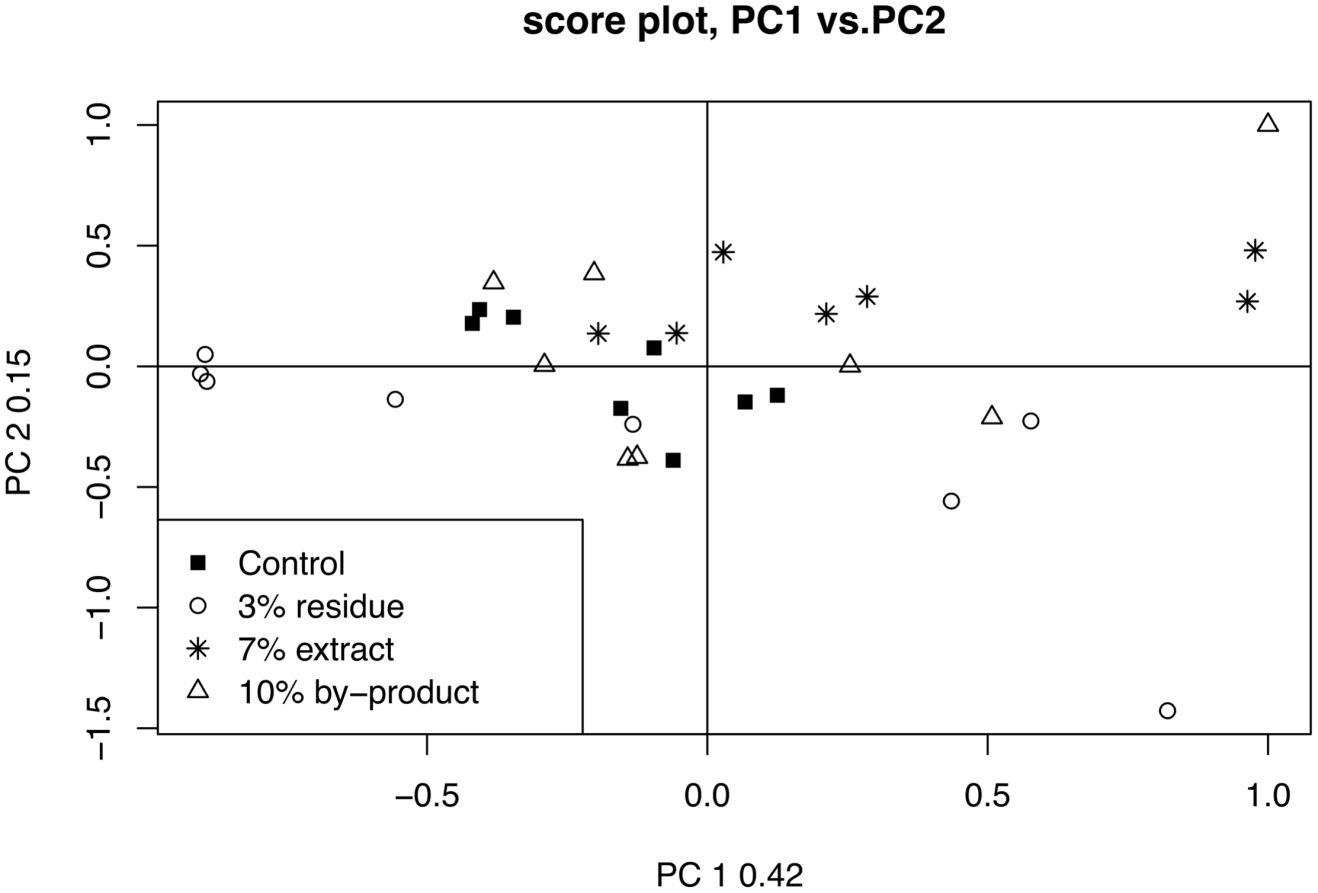
PCA analysis of onion mice data. The onion mice data matrix is Pareto scaled and centered. There are no clear trends that follow the onion intake percentage present in the PCA results, this matches the results of Winning et al. [37].

Corresponding with the original analysis by Winning et al. a principal component analysis (PCA) is performed. The feature data matrix is Pareto scaled and centered. The results of the PCA analysis, as presented as a score plot in Fig 7, are analogous to those of [37]: there are no obvious and consistent group trends that follow increases in onion intake.

#### Locating possible biomarkers with ease

**Fig 8:**
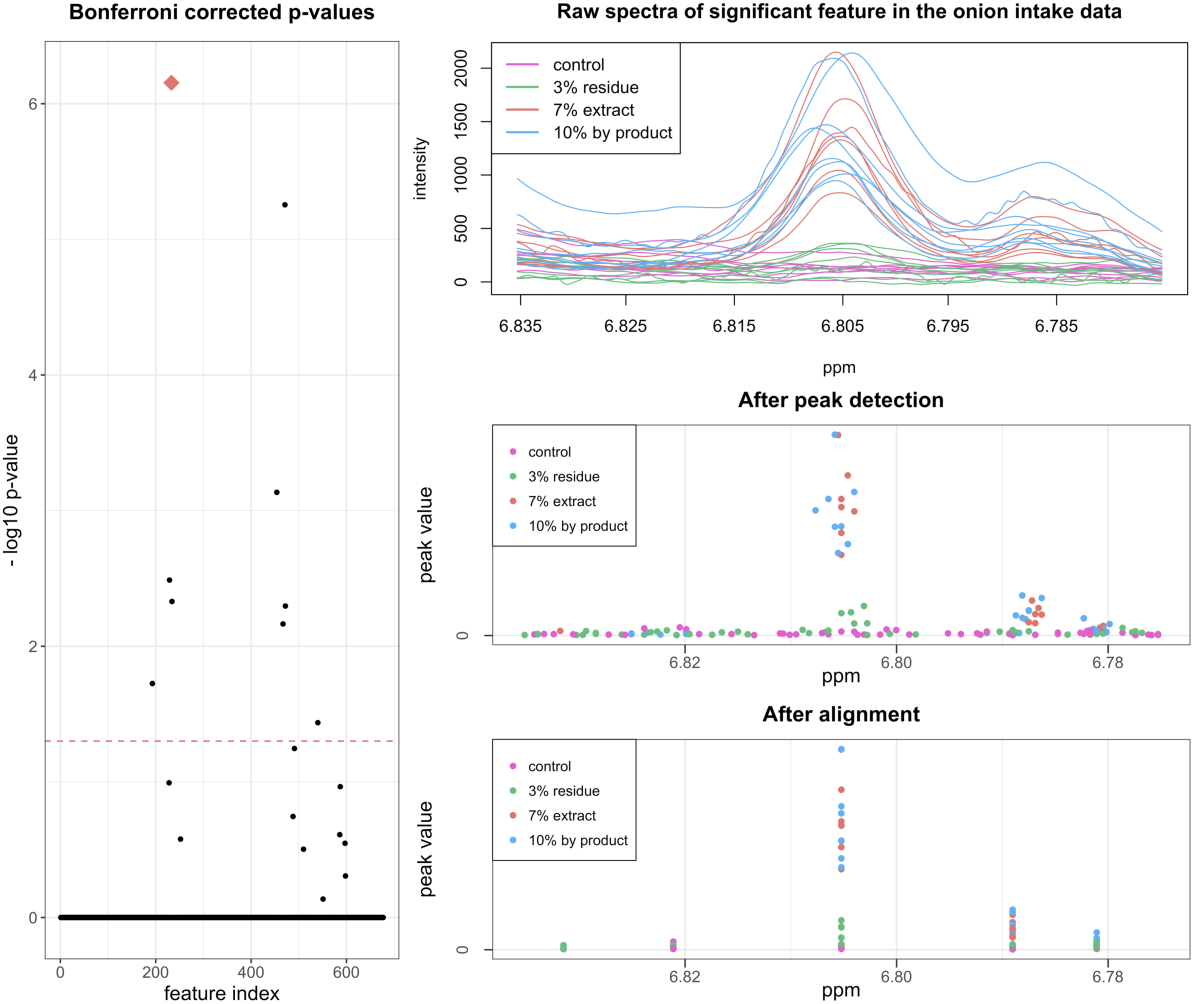
Differential analysis results of onion intake in mice data. (Left) the Bonferroni corrected p-values for the features resulting from the differential analysis and (right) one of the features with a significant p-value (indicated with the blue diamond on the left image): (top) raw spectra, (middle) data after peak detection and (bottom) data after grouping.

From this point onwards the merit of the wavelet based analysis becomes more obvious. Winning et al. resort to interval partial least squares (iPLS) and interval extended canonical variate analysis (iECVA). After careful cross validation, these methods lead to intervals that have to be checked manually for interesting peaks. The new speaq 2.0 workflow allows a quicker and more straightforward analysis. The constructed feature matrix is processed with the differential analysis method. In this case there exists a numerical relationship between all the groups (i.e. the percentage of onion in the diet), which is directly supported by the new speaq 2.0 differential analysis based on linear models. Each feature receives a Bonferroni corrected p-value assigned indicating how well the feature corresponds to the increasing onion concentration. The distribution of uncorrected p-values is depicted in S7 Fig. The corrected p-values are shown in Fig 8 along with an excerpt of one of the significant peaks. In total, 9 peaks were found to be significant. The 9 significant peaks can be used to search HMDB to find the possible biomarkers related to onion intake.

#### Identifying the biomarkers

**Fig 9:**
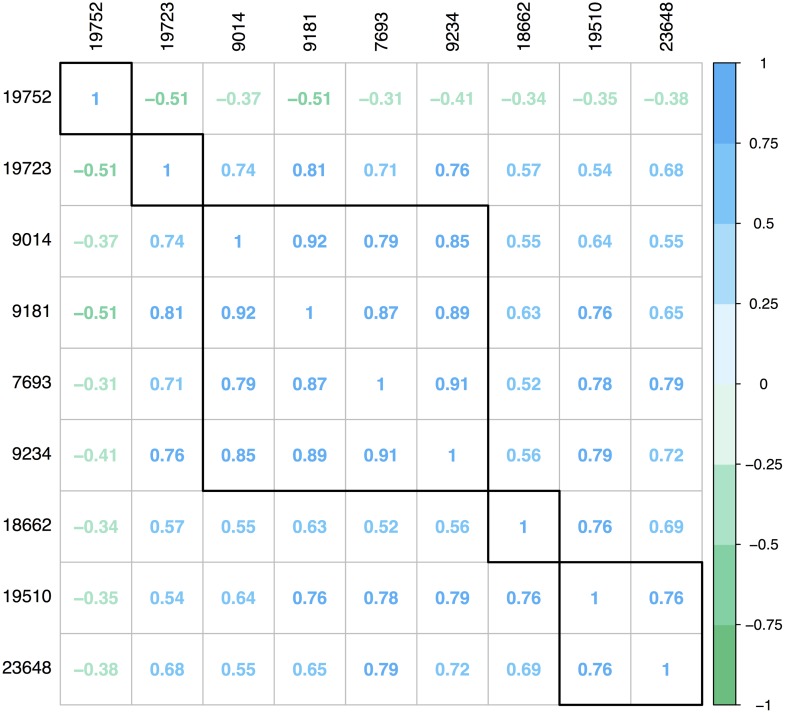
Correlation analysis of significant peaks. The significant peaks, which are indicated by their peakIndex value, are clustered based on their Pearson correlation. The group of four peaks correspond to the 3-hydroxyphenylacetic acid biomarker, peak nr. 19723 corresponds to the dimethyl sulfone biomarker. Both biomarkers are also identified in the original analysis paper [37], but with only one peak for the first biomarker.

Merely submitting all peak ppm values to HMDB will not produce the correct outcome, as HMDB expects all peaks to correspond to a single metabolite. To avoid submitting peaks from multiple metabolites to an HMDB search, a correlation-based clustering step is performed on the highly significant peaks. The result from this clustering, based on peak intensity correlations, is displayed in Fig 9. The significant peaks are grouped into 5 clusters, where the minimal Pearson correlation between any two peaks in the same cluster is higher than 0.75. These peak clusters are used to search HMDB (tolerance ± 0.02), by submitting the ppm values of the peak groups within a cluster. When submitting the cluster of 4 peaks, the top hit is 3-hydroxyphenylacetic acid (HMDB00440) with a Jaccard index of 4/9. This molecule is also identified in the original paper as a biomarker for onion intake. However, in the original paper this is done only by looking at a small region around 6.8 ppm, as compared to the speaq 2.0 analysis which yields peaks in multiple ppm regions that can be used for identification. The peak with index 18662 can actually also be assigned to 3-hydroxyphenylacetic acid (raising the Jaccard index to 5/9 upon also submitting this peak to HMDB). When the cluster that only contains peak 19723, with corresponding ppm value of 3.1558, is submitted to HMDB the top hits are dimethyl sulfone and 9-methyluric acid, both with a Jaccard index of 1/1. These results match those from the original paper where dimethyl sulfone (HMDB04983) is identified as a biomarker for onion intake. Raw spectra of the main peaks for both biomarkers are shown in S8 Fig.

#### The other peaks explained

The other peaks found cannot be identified querying HMDB. The peak with index 19510 is somewhat absorbed in the background. The peak with index 23648 ends up in a cluster with non-significant peaks that are assigned to ethanol within HMDB, when the correlation procedure is run on the entire dataset. As HMDB does not assign the 23648 peak to ethanol, this may indicate that this is a derivative or a byproduct of ethanol. The peak with index 19752 is actually a peak in the tail of the large peak of one of the identified biomarkers, namely dimethyl sulfone. The fact that this peak is significant is caused by an artifact of the wavelet based peak detection since it considers the tail of the large dimethyl sulfone peak as the baseline for the small peak. So when the dimethyl sulfone peak is larger, the baseline for the small peak is also larger and therefore the peak diminishes. This is also the reason why this peak is anti-correlated with the dimethyl sulfone peak.

#### Comparison with MetaboAnalyst

**Fig 10:**
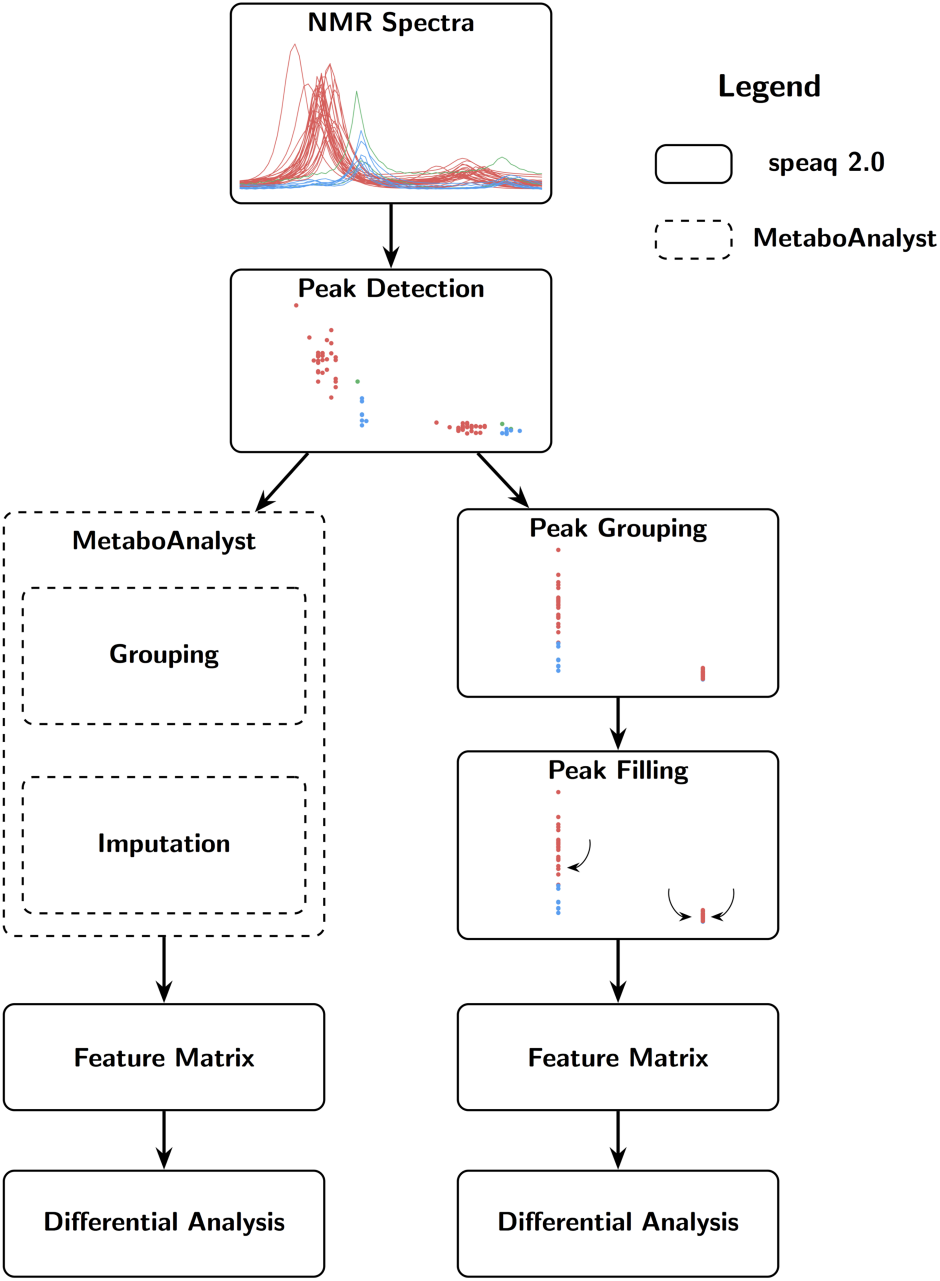
Workflow for comparing the results of MetaboAnalyst with speaq 2.0.

The MetaboAnalyst [12] platform is widely used for the analysis of metabolomics data. The processing of NMR data is also possible, provided the NMR data are supplied as a peak list or as binned data. Since Winning already used the binning approach, we will compare the results of MetaboAnalyst when peak data is supplied. This means the grouping step is performed in MetaboAnalyst thereby allowing the comparison to the speaq 2.0 grouping method. See Fig 10 for a visual representation of which steps of the workflow are different. The grouping method performed in MetaboAnalyst uses a moving window to group peaks together. The window is 0.03 ppm wide and moves with steps of 0.015 ppm according to the documentation. If more than one peak per sample is detected in a single group the intensities of these peaks are summed together. After pre-processing with MetaboAnalyst the data matrix (245 features) is extracted and processed with the differential analysis function. So again each column in the MetaboAnalyst matrix gets a (Benjamini-Hochberg corrected) p-value assigned to indicate how well this feature corresponds to the increasing onion diet. The results are presented in Table 2. For every highly significant feature in the MetaboAnalyst data, there is at least one highly significant feature from the speaq 2.0 analysis. The difference between both methods is the lower resolution of MetaboAnalyst, as it sums close peaks together. This approach effectively removes a source of information contained in the data as multiplets can aid in the identification of compounds.

**Table 2:** Comparison between MetaboAnalyst and speaq 2.0 for the onion intake in mice dataset.

MetaboAnalyst			speaq 2.0		
group ppm	p-value		group ppm	p-value	
3.479	3.48 E-07	**	3.480	2.44 E-04	**
6.806	6.78 E-07	**	6.805	7.01 E-07	**
			6.821	3.02 E-01	
6.782	2.32 E-05	**	6.789	8.42 E-04	**
			6.781	6.45 E-02	
			6.769	7.47 E-01	
			6.763	8.59 E-01	
3.156	2.39 E-05	**	3.156	2.78 E-06	**
			3.147	8.42 E-04	**
			3.163	9.15 E-01	
6.855	1.42 E-04	**	6.856	8.13 E-04	**
			6.832	6.38 E-01	
			6.841	9.54 E-01	
2.774	6.40 E-03	**	2.781	5.67 E-03	**
			2.773	5.08 E-01	
1.324	1.07 E-02	*	1.325	9.04 E-03	**
			1.341	1.75 E-02	*

This table shows the significant groups of MetaboAnalyst and the corresponding peaks found by speaq 2.0 within the same bins. For every highly significant feature in the MetaboAnalyst dataset there is at least one highly significant feature in the speaq 2.0 results. The difference is however quite clear. Whereas MetaboAnalyst constructs a feature by summing intensities of peaks within small intervals, the speaq 2.0 method uses all peaks. Note that the p-values in this table are Benjamini-Hochberg corrected (the default in speaq 2.0). [**p < 0.01; * *p* < 0.05].

## Concluding remarks

We present an easy way of converting 1D NMR spectra (or other 1D spectra) to peak data by using wavelets for peak detection. This wavelet based method performs better than binning or other spectra summarizing methods as the dimension of the dataset is greatly reduced with little to no loss of information, while requiring no user intervention. After the wavelet based step the peaks are grouped via a hierarchical clustering method. These groups of peaks are called features. The features can easily be analyzed with a myriad of statistical techniques or data mining approaches. Our method has been implemented in an entirely new version of the existing speaq R package which offered the CluPA algorithm for aligning spectra. This package now provides an entire solution for easy 1D NMR data analysis without the need for binning. Each step in the workflow is available as a single function. Thus, analysis pipelines can be constructed easily and with little additional user interaction, fostering improved research reproducibility and shareability.

Besides the possibility to perform a complete standalone analysis, our method can also be used in tandem with other commonly used tools that rely on summarized spectra. Specifically, it can be used to quickly and efficiently produce a high quality peak list. Such a peak list is the starting point of an analysis with for example the often used MetaboAnalyst [12].

The data processed in this article came in a matrix format with ppm values and intensities. Other proprietary software or open-source frameworks are thus needed if only the raw NMR Free Induction Decay signal (FID) is available and conversion to the frequency space is needed. Optional pre-processing steps on the raw FID signal like zero-filling, apodization, and phase-shifting have to be performed prior to employing speaq 2.0, if they are desired. These pre-processing steps are on the road-map for future developments.

We expect the introduced method to be especially useful for processing NMR spectra from large cross-platform experiments that combine NMR and LC-MS. Often software packages like XCMS [5] are used to process LC-MS data. These open source packages also employ the standard paradigm of peak-picking, grouping, etc. so the integration of data or results should be facilitated with this framework. The method in itself also has merit as is clearly demonstrated in the case of the onion intake in mice data. The analysis is fast, sensitive to both small and large peaks and user-independent. Also, when comparing the results we obtained to the work presented by Winning et al. [37], our analysis required less user interaction and yields more peaks in the end that can be used to identify the possible biomarkers, resulting in an improved confidence in the results.

The user-friendliness of speaq 2.0 should also allow people with little experience in R to use the package. Also, it can serve as an attractive option for researchers interested in switching from closed, proprietary software to open-source, especially if the goal is to speed up analysis, improve reproducibility and increase control over workflows and algorithms.

## Availability and future directions

speaq 2.0 is distributed through the existing speaq R package to provide a complete solution for NMR data processing. The package and the code for the presented case studies are freely available on CRAN (https://cran.r-project.org/package=speaq) and GitHub (https://github.com/beirnaert/speaq). Future directions will aim to provide compatibility with the open source nmrML (http://www.nmrml.org) format and to improve on the identification part by combining our approach with Statistical Total Correlation Spectroscopy (STOCSY) [38].

## Supporting information

S1 Appendix Grouping algorithm details. (PDF)

S2 Appendix Simulated data based comparison of speaq 2.0 vs alignment algorithms. (PDF)

S3 Appendix Silhouette values and the SilhouetR function. (PDF)

S1 Fig Wine data PCA plot. The PCA score plot shows that Principal Component 2 clearly indicates a difference between red, white and rosé wines.
(PNG)

S2 Fig Difficulties arise in crowded spectra. When many peaks are present in a small region, it is not clear which peaks correspond to each other. The speaq 2.0 method, based on finding peaks and subsequently grouping, performs similar or better compared to the other methods as it sees all peaks and tries to group closer ones together. The CluPA algorithm uses landmark peaks and therefore simply tries to align the largest ones together, which is not correct in this case. Lastly, the icoshift algorithm tries to align the spectra based on correlations but the result in this crowded region is also not satisfactory.
(PNG)

S3 Fig A sample with unique behavior causes issues. In the region around 5.43 ppm there appear to be two small peaks in all samples. A single sample of red wine has two additional large peaks around the 5.40 ppm region. Every method performs poor in this case: both icoshift and CluPA (speaq v1—v1.2.3) align the two large peaks with the group of small peaks. The CluPA algorithm does this by shifting the entire region to the right, this results in the two small peaks of these spectra to be shifted to the right of the small peaks group around 5.43 ppm. The icoshift algorithm on the other hand introduces some strange artifacts and the two small peaks are gone all together. The speaq 2.0 algorithm deletes one of the large peaks in the grouping step, which it often does if multiple peaks from the same sample are present in one group. This problem is usually mitigated by the peak filling step but in this case it is not.
(PNG)

S4 Fig Between-sample shifts that are larger than between-adjacent-peaks shifts can cause problems. In this case both raw spectra methods perform as expected whereas the speaq 2.0 method does not. Initially peaks are wrongly grouped together. This problem is however detected by the optional *SilhouetR* function in speaq 2.0 which calculates the silhouette values for each group. After the appropriate correction the results are as expected.
(PNG)

S5 Fig Raw spectra of the onion intake in mice data. (PNG)

S6 Fig speaq 2.0 workflow applied to the onion intake in mice data [37]. (A) Onion intake in mice peak data after grouping and filling. The gap in the raw data is clearly visible: this data was removed by the study authors because of insufficient water suppression. (B) Excerpt of peak data pre-grouping. (C) Excerpt of peak grouped data.
(PNG)

S7 Fig Distribution of uncorrected p-values. The possible biomarker signals are clearly present on the left as an increase in frequency over the otherwise uniform distribution.
(PNG)

S8 Fig NMR spectra of biomarkers identified by speaq 2.0. Main peaks of both biomarkers from the onion intake in mice data [37]. (Top) dimethyl sulfone and (bottom) 3-hydroxyphenylacetic acid.
(PNG)

S9 Fig Spectral alignment algorithms can introduce artifacts. The results of spectral alignment algorithms is not always optimal when dealing with severely shifted spectra. This is illustrated here for the simulated case vs control data (*m*_*i*_ is bimodal). The algorithms can introduce artifacts, i.e. misalign or overcorrected spectra, which affect the following processing steps (e.g. binning).
(PNG)
