# Supplementary material for: speaq 2.0: A complete workflow for high-throughput 1D NMR spectra processing and quantification
Source: PLoS Comput Biol. 2018 Mar 1;14(3):e1006018. doi: 10.1371/journal.pcbi.1006018 (PMC5849334; doi:10.1371/journal.pcbi.1006018)
Supplement: S1 Appendix — (PDF) [file pcbi.1006018.s001.pdf]

S1 Appendix

Grouping algorithm details.

The grouping algorithm receives the data after the peak detection and tries to group the peaks. Grouped peaks receive a common group index, but keep their individual peak data like ppm, SNR, peakValue, etc. Some peaks may not be assigned to a group after this procedure. However, this is not a major issue, as not all peaks are equally relevant and some may be artifacts. Nonetheless, if a removed peak is in fact a valid peak, this is mitigated by the peak filling step which follows the grouping. The grouping algorithm works according to the following scheme (note that the full code can also be inspected on CRAN or GitHub):

1. Define window of interest:
   (a) take the standard window width of 100 (adjustable) measurement points and vary it by 10% left and right and check if any peaks emerge or disappear. This is to limit the possibility of splitting a group when selecting a window.
   (b) if there are peaks added or disappearing when varying the window size, then the window width is multiplied with a factor 2 and the process of varying the window width starts again (if this keeps going on, the window size is decreased again but in smaller steps).
2. The peaks in the window are divided into groups by using a hierarchical cluster tree in a top-down fashion, meaning that all peaks start in the same group. The ppm values of the peaks are used to build the distance matrix (Euclidean distance) which is used to build the tree, then:
   (a) whenever a group is split up from the rest of the tree, this group is checked for its proportion of duplicated peaks (multiple peaks from the same sample in a single group).
   (b) if few duplicates are present (default 25%), this subtree is marked as a single group and the center peakindex is assigned to all members,
   (c) if no group with few duplicates is present (anymore), the algorithm moves to the next lower level in the tree.

3. The groups are checked for duplicate peaks, if duplicate peaks are present then these are removed: because of the sensitivity of the wavelet based peak detection very small peaks in the tails of larger ones can be detected. Since these are very close together they will automatically be assigned to the same cluster and there is no problem with removing the duplicated peak. The wavelet based peak detection allows the identification of the unwanted peak not only by the peak ppm, but also by the signal-to-noise ratio, as this small bump will not have the same SNR as the rest of the peak population in the group. Therefore the Gower distance matrix is calculated [29] with peak ppm and SNR and the duplicated peak furthest away from the group is deleted. The Gower distance is in this case preferred as ppm and SNR vary on different scales.

4. The last steps consist of a verification steps:

   (a) a sliding window verification step: sliding window based approaches often result in errors as there is manually check whether the edge of a window is not cutting a group in two halves. Groups on adjacent sides of the sliding window edges are merged and regrouped one final time. Although measurements have been taken in the first steps by inflating (or deflating) the window and all neighbors have been regrouped already in the previous step, should a group still be badly grouped this step should fix it.

   (b) an overall grouping verification step: in rare cases, it might occur that a group is nonetheless split up into multiple parts. To verify this, the Jaccard index of all neighboring peak groups is calculated. The Jaccard index is the division of the common samples by all samples. If the Jaccard index is low, we might assume that these two groups might be one and the same and they are reanalyzed by placing them in one cluster. If the groups were in fact one and the same, they will be placed within the same cluster and otherwise they will be kept separate in two distinct clusters.
