# Supplementary material for: speaq 2.0: A complete workflow for high-throughput 1D NMR spectra processing and quantification
Source: PLoS Comput Biol. 2018 Mar 1;14(3):e1006018. doi: 10.1371/journal.pcbi.1006018 (PMC5849334; doi:10.1371/journal.pcbi.1006018)
Supplement: S2 Appendix — (PDF) [file pcbi.1006018.s002.pdf]

S2 Appendix

Simulated data based comparison of speaq 2.0 vs alignment algorithms. The simulated spectra, $\mathbf{S}_i$, are constructed by combining the spectra of metabolites 3-Hydroxyphenylacetic acid (HMDB0000440) and 3,4-Dihydroxybenzeneacetic acid (HMDB0001336), respectively indicated with $\mathbf{S}_1$ and $\mathbf{S}_2$. This combination is done as follows:

$$\mathbf{S}_i = a_i \cdot [m_i \cdot \widetilde{\mathbf{S}}_1^i + (1 - m_i) \cdot \widetilde{\mathbf{S}}_2^i] + \varepsilon \tag{1}$$

with a_i an amplitude factor chosen at random in a specific interval (see Table 1) and m_i a mixing factor chosen at random in $[0, 1]$ or fixed in case of only one metabolite spectrum. The $\sim$ indicates that the spectra are shifted left or right. The amount of shift i is chosen at random in a specific interval (see Table 1). ε is an additive noise term with a maximum amplitude of 1% of the largest intensity in the base spectra.

Table 1. The parameters for each individual simulated dataset.

Nr.	m_i	a_i	i (ppm)	Comment
1	1	$[0.2, 2.0]$	$[-0.002, 0.002]$	1 spectrum with small shifts
2	1	$[0.2, 2.0]$	$[-0.004, 0.004]$	1 spectrum with large shifts
3	$[0, 1]$	$[0.2, 2.0]$	$[-0.004, 0.004]$	2 spectra with large shifts
4	$(0.25, 0.75) \pm 0.15$	$[0.8, 1.2]$	$[-0.004, 0.004]$	case vs control (m_i bimodal)

The indication whether a shift is small or large is relative and depends on the distances between adjacent peaks. A shift indicated as large corresponds to a shift that will cause adjacent peaks to overlap or even switch position. A shift indicated with small corresponds to shift that only allow neighboring peaks to partly overlap.

The ground truth for the simulated data is provided by HMDB [34] in the form of peak lists. These lists provide the locations (in ppm) of the peaks that are associated with the metabolites. With these peak locations we can quantify the speaq 2.0 grouping algorithm by calculating external indices for cluster validation [1^S]. Specifically, the adjusted RAND index and the Jaccard index are used, see Table 2. These indexes quantify how well a grouping or clustering algorithm performs based on externally provided ground truth clusters (the peak lists). An index of 0 indicates that nothing is grouped together, an index of 1 indicates that all elements are clustered correctly.

Note that different metrics need to be used to quantify the performance of the spectral alignment techniques (icoshift and CluPA) compared to the speaq 2.0 peak based approach. This stems from the fact that these alignment algorithms perform an intrinsically different task compared to the peak clustering of speaq 2.0. Assessing the quality of spectral alignment tools is often done visually [20, 27]. However, in this case we can determine what the end result should be since we can easily remove the introduced shift i to produce an unshifted dataset. To assess the quality of the alignment we can calculate the correlation of each spectrum to a standard spectrum that is composed of either 1 unshifted base spectrum ($m_i = 1$, $a_i = 1$ and $i = 0$), or the equal combination of both unshifted base spectra ($m_i = 0.5$, $a_i = 1$ and $i = 0$). Important to note is that the random noise is removed from these spectra before the alignment and correlation calculation. In this case the correlation will give an indication of the alignment quality without being influenced by the (lack of) correlation between the noise. See Table 3 for the results.

Table 2. Grouping performance of speaq workflow.

Dataset	adjusted Rand index	Jaccard index
1	1.000	1.000
2	0.955	0.921
3	0.719	0.798

Table 3. Correlation metrics of the alignment algorithms results.

Dataset	average shifted corr	average unshifted corr	average CluPA corr	average icoshift corr
1	0.911	1.000	1.000	0.999
2	0.762	1.000	1.000	0.999
3	0.741	0.936	0.943	0.939

Several things can be noted in Table 2:

1. The icoshift and CluPA algorithms perform perfectly in the first two simulated datasets. The reason for this is that these algorithms are in part based around correlations between the spectra that are to be aligned. And in case there is only 1 base spectrum which is shifted randomly and multiplied with a random factor,

there will be always at least one shift correction for which the correlation is exactly 1.

2. The performance of our method decreases with increasing spectral complexity. This is as expected since it becomes more difficult to group the peaks correct when more and more peaks start to overlap or even switch position.

3. The results of the last simulated dataset suggest that icoshift and CluPA perform better than the theoretical optimum. Specifically, the average correlation between the corrected spectra and the base spectrum is higher than the average correlation between the unshifted spectra and the base spectrum. This indicates that CluPA and icoshift overcorrect the shifts present in the data. Such a situation is clearly visible in S9 Fig. When post-processing this data with the binning approach a number of bins will contain peaks from different locations that do not belong together.

It is not straightforward to quantify the performance of the alignment algorithms objectively. The results in Table 2 indicate that the aligned spectra are highly correlated to the base spectrum. However, as shown in S9 Fig this does not always imply that the spectra are aligned correctly. Any misalignments will cause artifacts in the following processing step, which is usually binning. To investigate the effects of these artifacts and to objectively compare both our method to the raw spectra methods a case vs control dataset is simulated from a mixture with a bimodal distribution. Specifically m_i is drawn from 2 normal distributions with respective means of 0.25 and 0.75, both with standard deviations of 0.15. This case vs control simulated data set is analyzed in the main manuscript text.

Supporting References

1^S. Dudoit S, Fridlyand J. A prediction-based resampling method for estimating the number of clusters in a dataset. Genome biology. 2002;3(7):research0036.1.
