# Supplementary material for: speaq 2.0: A complete workflow for high-throughput 1D NMR spectra processing and quantification
Source: PLoS Comput Biol. 2018 Mar 1;14(3):e1006018. doi: 10.1371/journal.pcbi.1006018 (PMC5849334; doi:10.1371/journal.pcbi.1006018)
Supplement: S3 Appendix — (PDF) [file pcbi.1006018.s003.pdf]

S3 Appendix

Silhouette values and the SilhouetR function. After a grouping step it can be useful to calculate the silhouette values [2S]. The silhouette value is a measure of how similar an element of that group is compared to its own group vs compared to other groups. The silhouette values range from -1 to 1, with higher values indicating that the element is more similar to its own group than to others. Groups with an on average low silhouette value could be wrongly grouped. An optional function in speaq allows users to calculate these silhouette values.

Supporting References

2S. Rousseeuw P. Silhouettes: a graphical aid to the interpretation and validation of cluster analysis. Journal of Computational and Applied Mathematics. 1987;20:53–65.
