# Supplementary figures and images for: speaq 2.0: A complete workflow for high-throughput 1D NMR spectra processing and quantification
Source: PLoS Comput Biol. 2018 Mar 1;14(3):e1006018. doi: 10.1371/journal.pcbi.1006018 (PMC5849334; doi:10.1371/journal.pcbi.1006018)

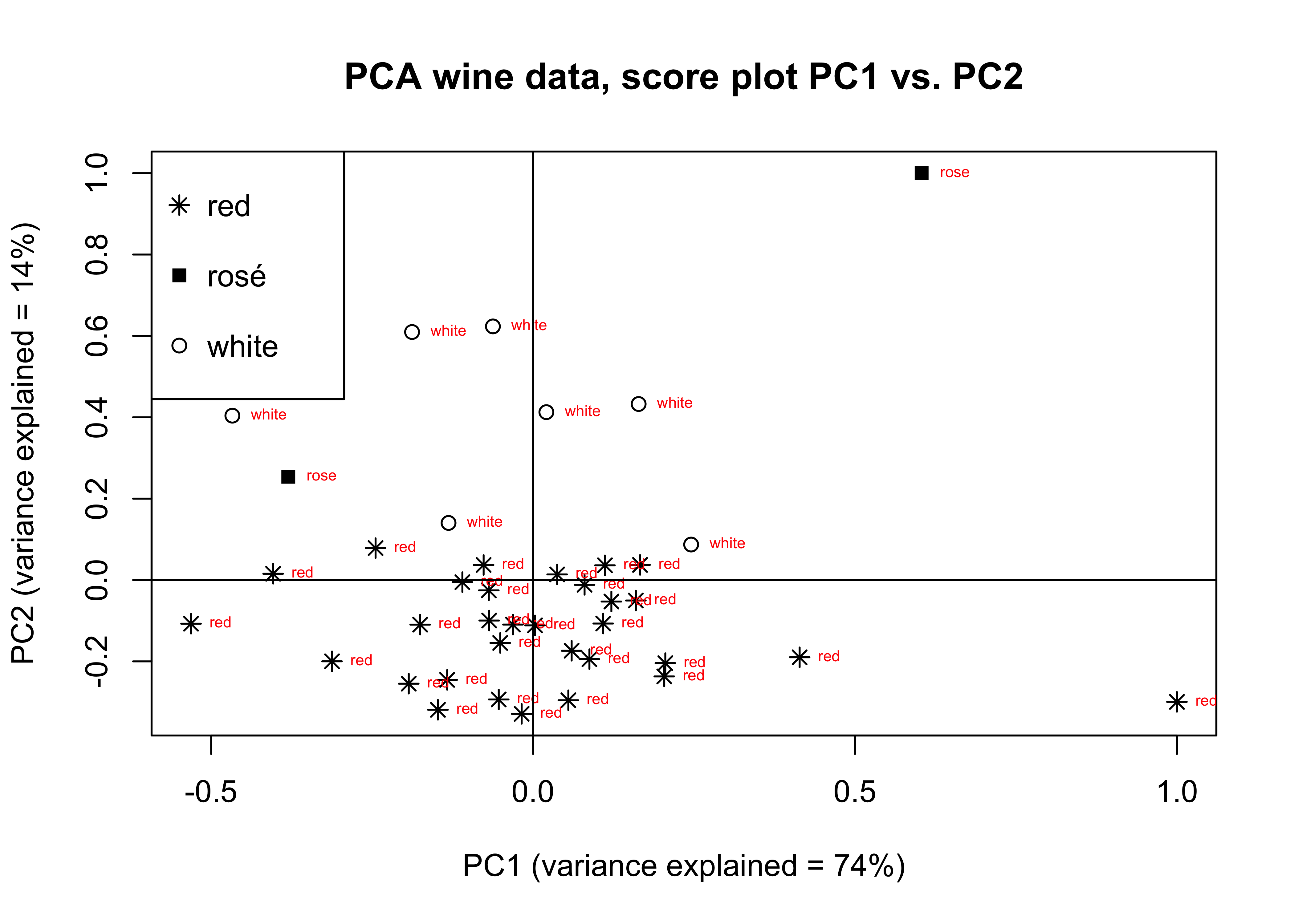

Supplement: S1 Fig — The PCA score plot shows that Principal Component 2 clearly indicates a difference between red, white and rosé wines. (PNG) [file pcbi.1006018.s004.png]

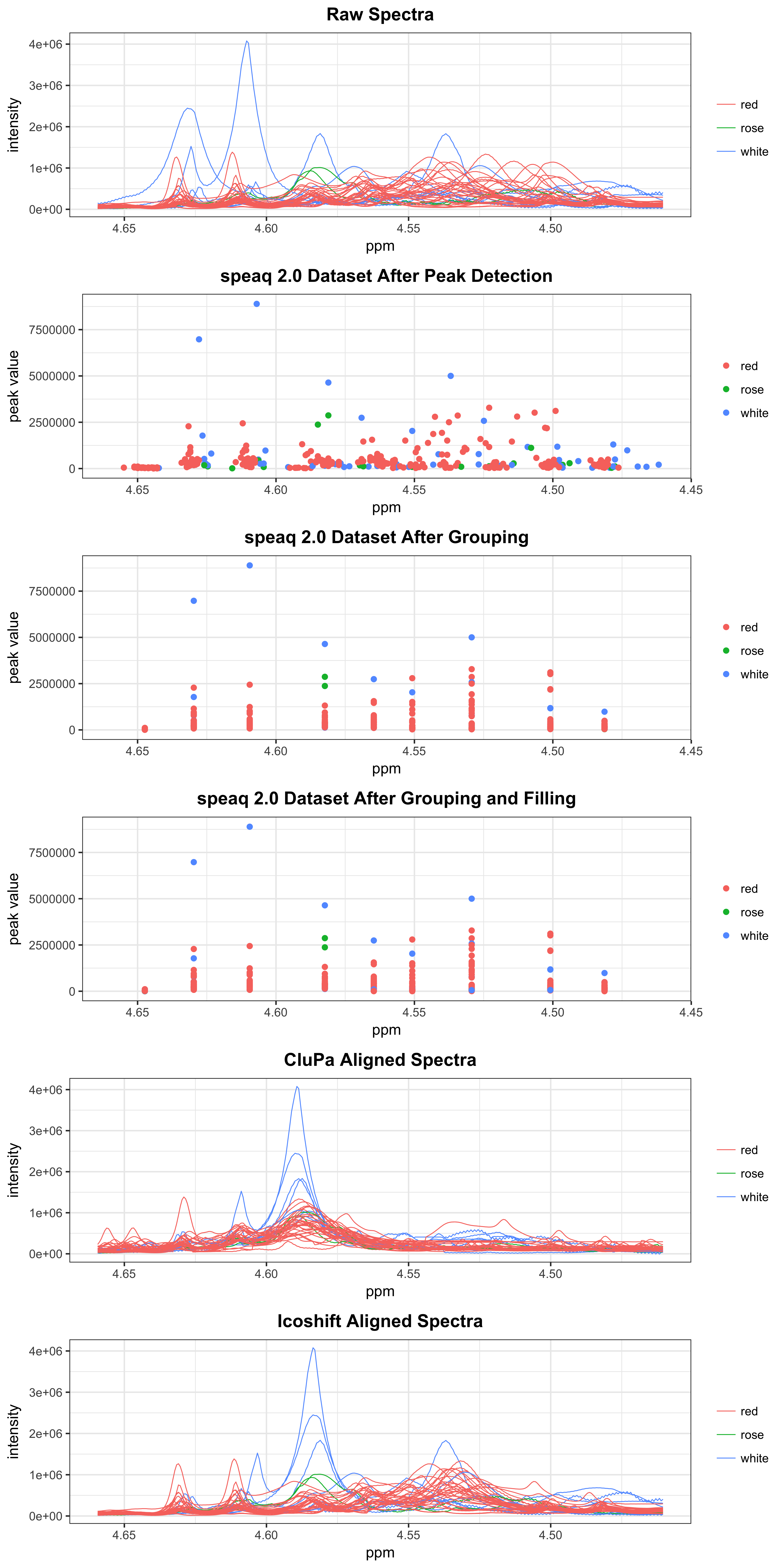

Supplement: S2 Fig — When many peaks are present in a small region, it is not clear which peaks correspond to each other. The speaq 2.0 method, based on finding peaks and subsequently grouping, performs similar or better compared to the other methods as it sees all peaks and tries to group closer ones together. The CluPA algorithm uses landmark peaks and therefore simply tries to align the largest ones together, which is not correct in this case. Lastly, the icoshift algorithm tries to align the spectra based on correlations but the result in this crowded region is also not satisfactory. (PNG) [file pcbi.1006018.s005.png]

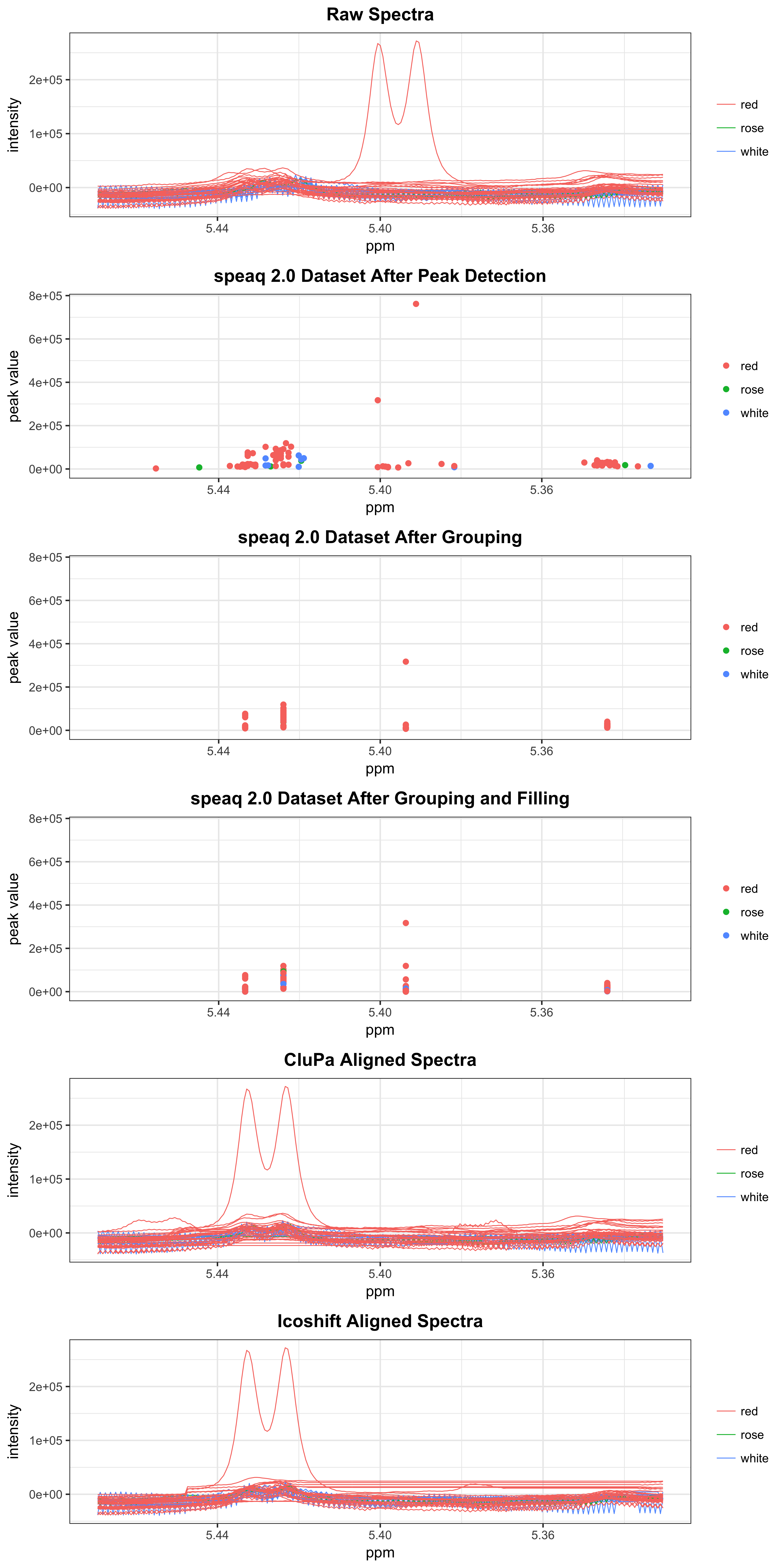

Supplement: S3 Fig — In the region around 5.43 ppm there appear to be two small peaks in all samples. A single sample of red wine has two additional large peaks around the 5.40 ppm region. Every method performs poor in this case: both icoshift and CluPA (speaq v1—v1.2.3) align the two large peaks with the group of small peaks. The CluPA algorithm does this by shifting the entire region to the right, this results in the two small peaks of these spectra to be shifted to the right of the small peaks group around 5.43 ppm. The icoshift algorithm on the other hand introduces some strange artifacts and the two small peaks are gone all together. The speaq 2.0 algorithm deletes one of the large peaks in the grouping step, which it often does if multiple peaks from the same sample are present in one group. This problem is usually mitigated by the peak filling step but in this case it is not. (PNG) [file pcbi.1006018.s006.png]

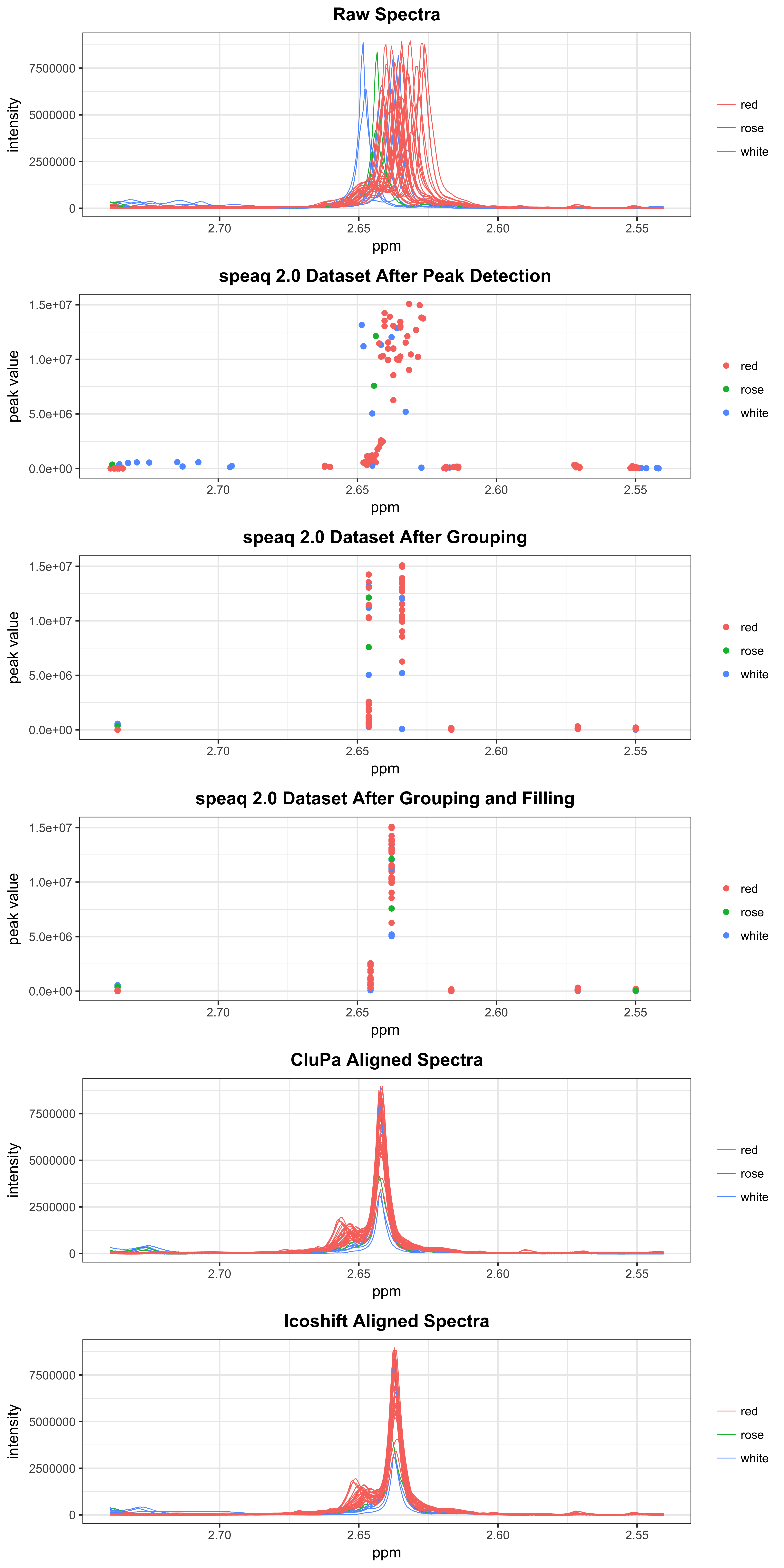

Supplement: S4 Fig — In this case both raw spectra methods perform as expected whereas the speaq 2.0 method does not. Initially peaks are wrongly grouped together. This problem is however detected by the optional SilhouetR function in speaq 2.0 which calculates the silhouette values for each group. After the appropriate correction the results are as expected. (PNG) [file pcbi.1006018.s007.png]

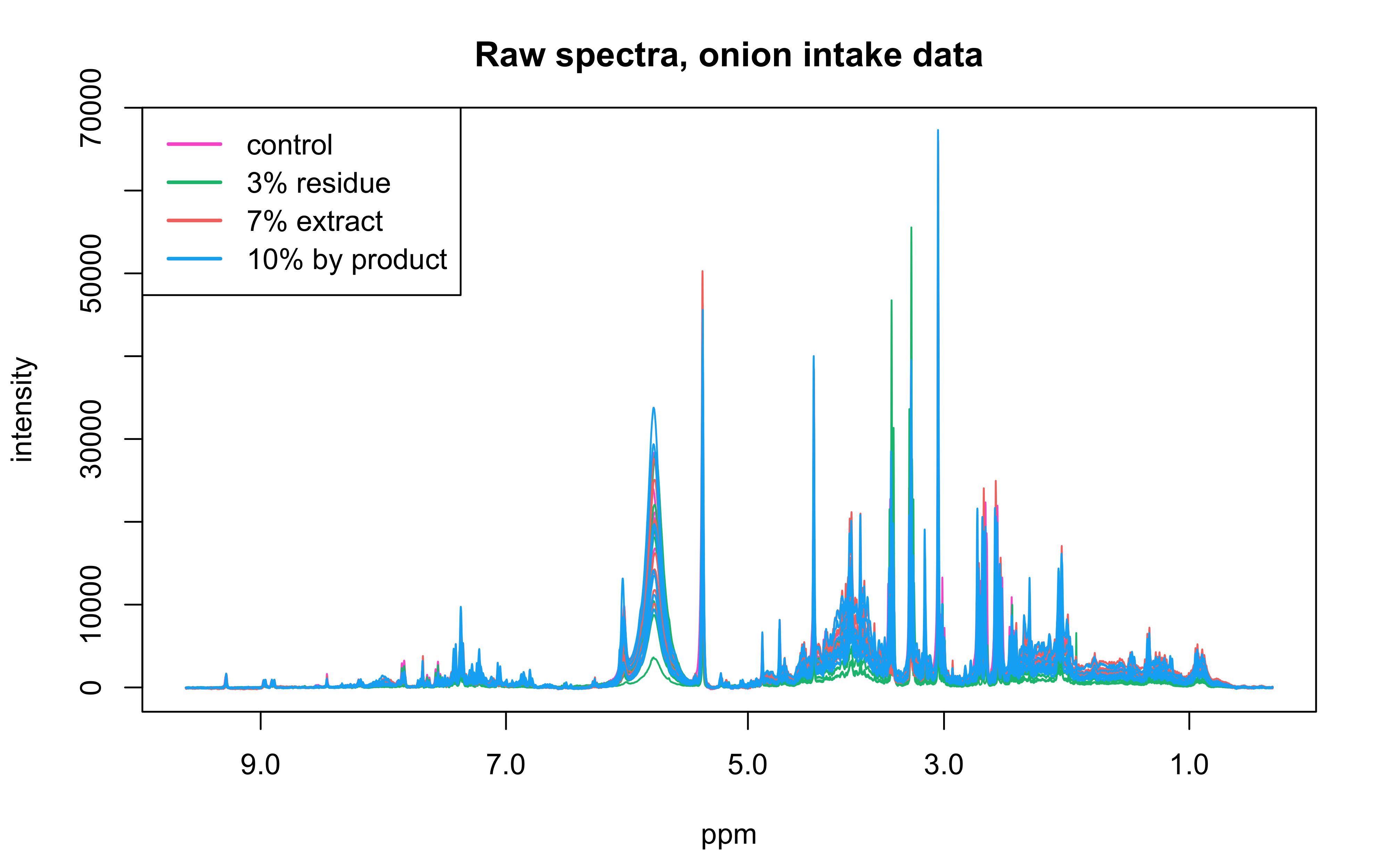

Supplement: S5 Fig — (PNG) [file pcbi.1006018.s008.png]

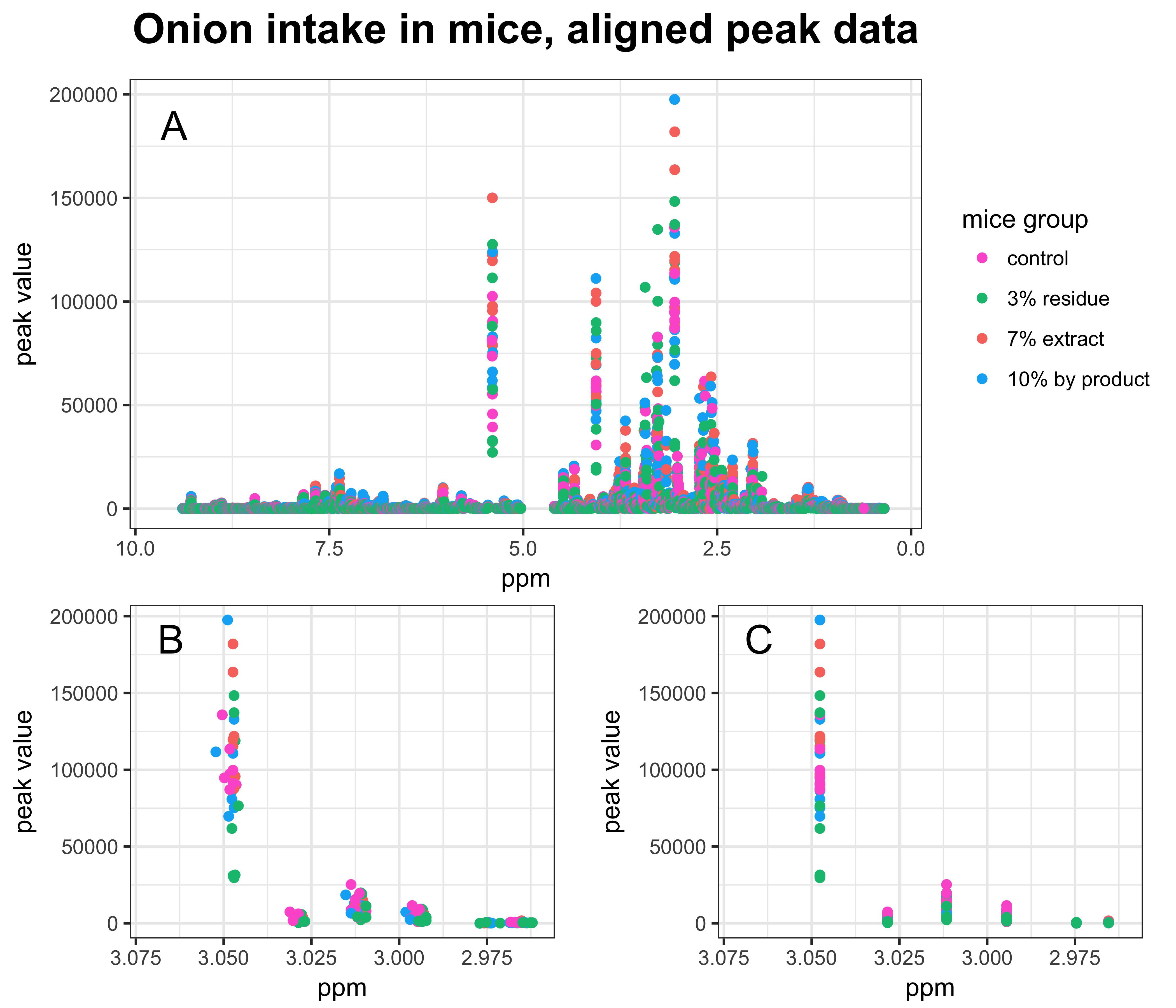

Supplement: S6 Fig — (A) Onion intake in mice peak data after grouping and filling. The gap in the raw data is clearly visible: this data was removed by the study authors because of insufficient water suppression. (B) Excerpt of peak data pre-grouping. (C) Excerpt of peak grouped data. (PNG) [file pcbi.1006018.s009.png]

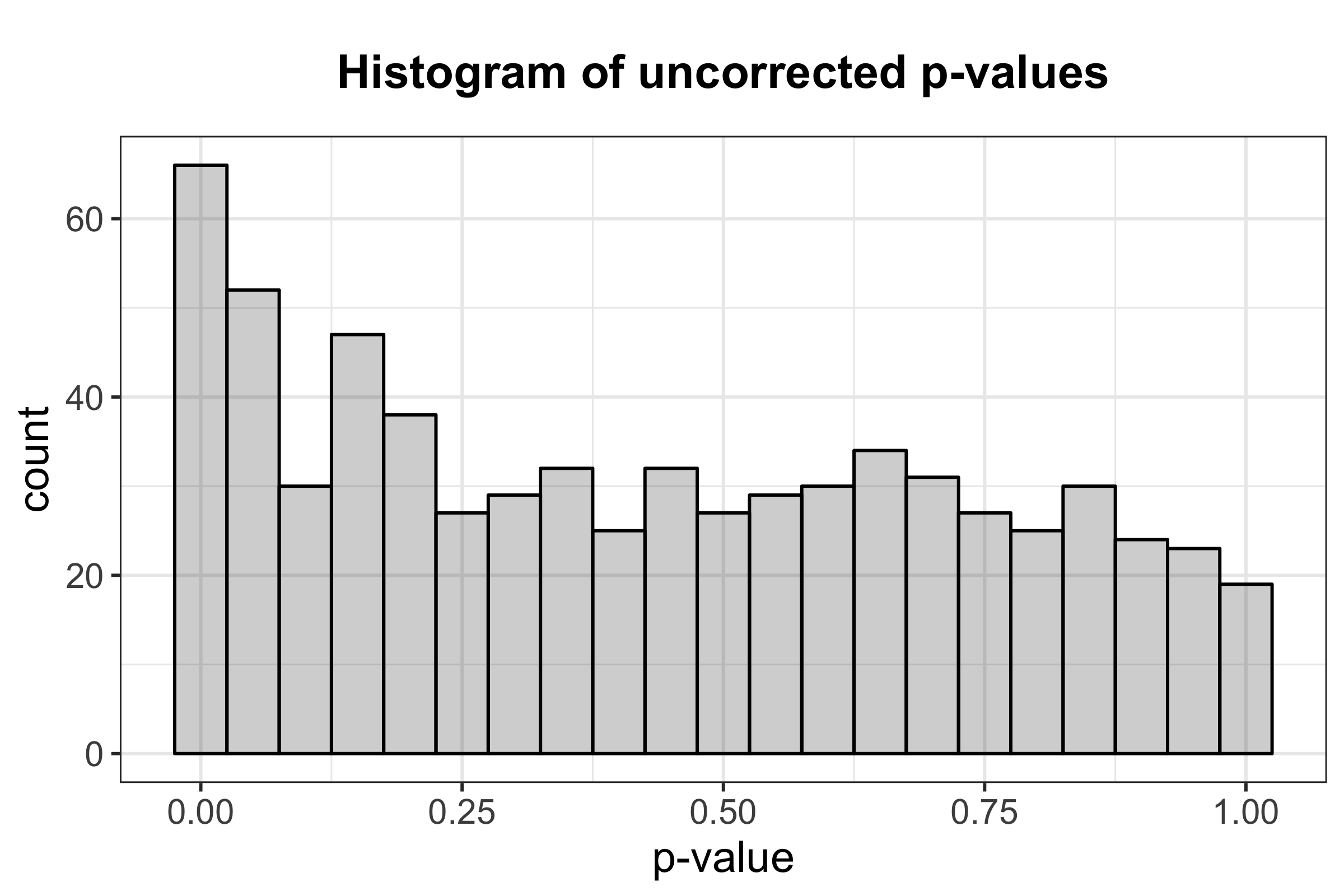

Supplement: S7 Fig — The possible biomarker signals are clearly present on the left as an increase in frequency over the otherwise uniform distribution. (PNG) [file pcbi.1006018.s010.png]

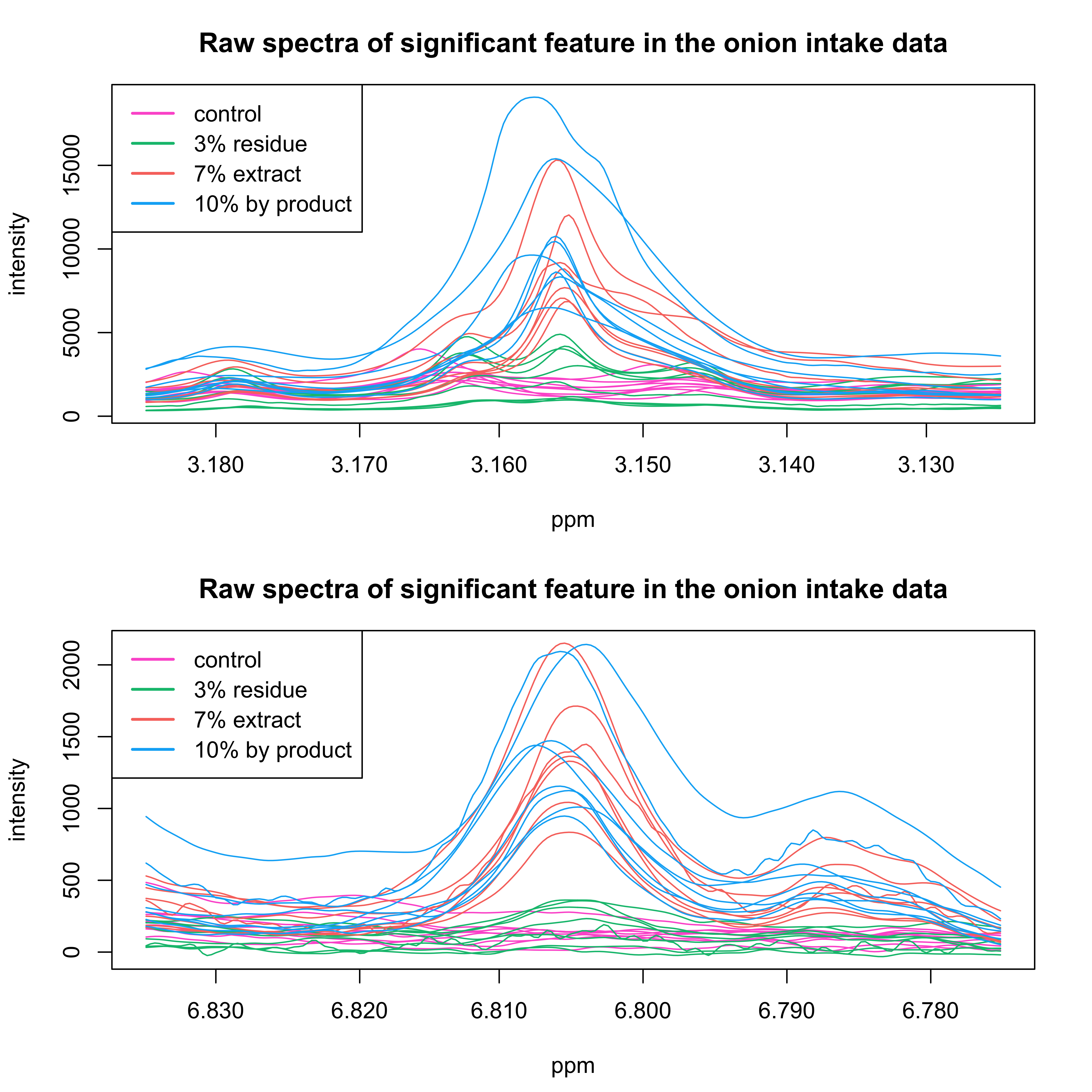

Supplement: S8 Fig — Main peaks of both biomarkers from the onion intake in mice data [37]. (Top) dimethyl sulfone and (bottom) 3-hydroxyphenylacetic acid. (PNG) [file pcbi.1006018.s011.png]

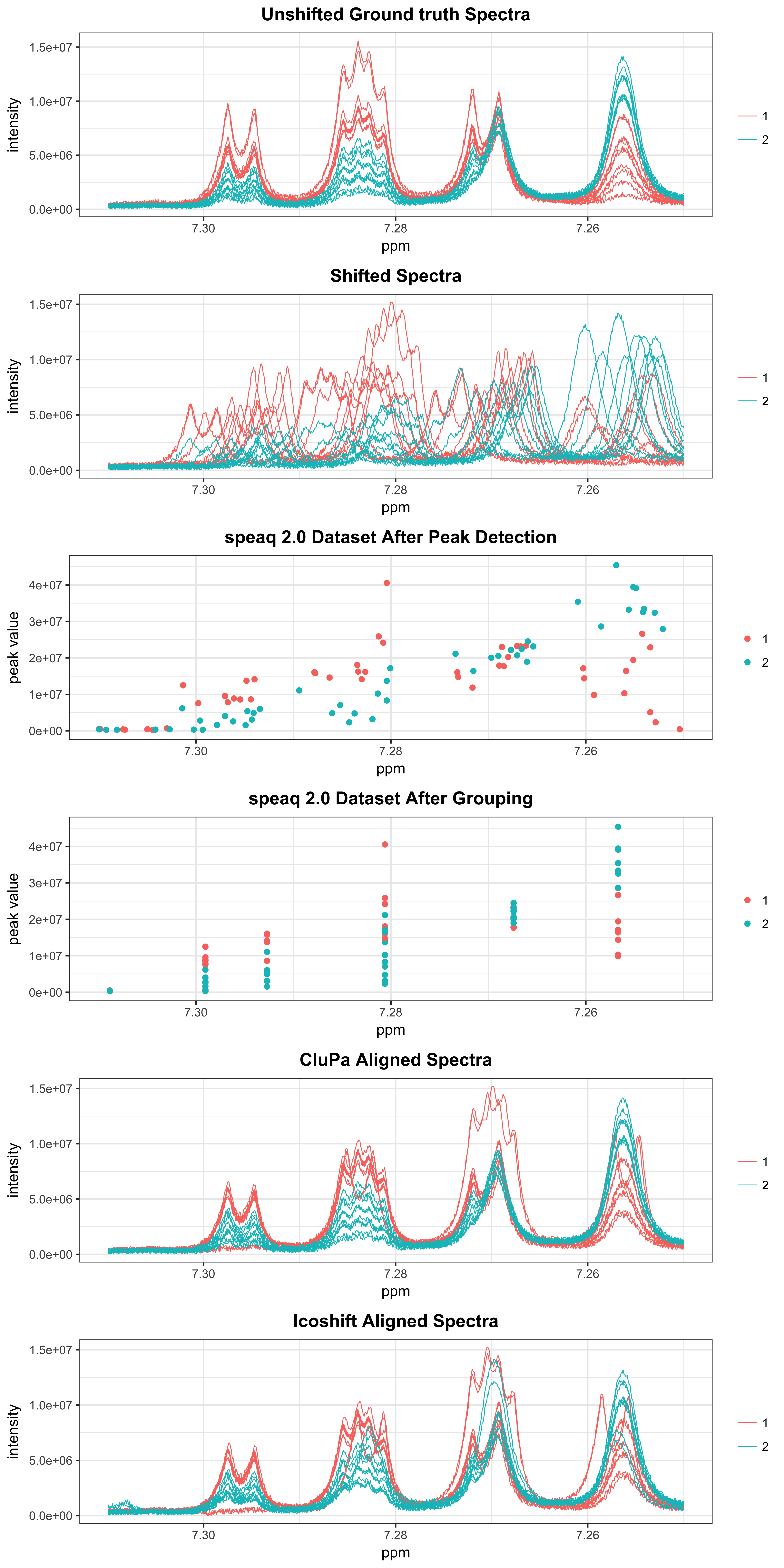

Supplement: S9 Fig — The results of spectral alignment algorithms is not always optimal when dealing with severely shifted spectra. This is illustrated here for the simulated case vs control data (mi is bimodal). The algorithms can introduce artifacts, i.e. misalign or overcorrected spectra, which affect the following processing steps (e.g. binning). (PNG) [file pcbi.1006018.s012.png]
